# Inflammation and Cardiovascular Disease Associated With Hemodialysis for End-Stage Renal Disease

**DOI:** 10.3389/fphar.2022.800950

**Published:** 2022-02-10

**Authors:** Yinghui Wang, Lu Gao

**Affiliations:** Department of Cardiovascular Centre, The First Hospital of Jilin University, Jilin, China

**Keywords:** inflammation, hemodialysis, cardiovascular disease, chronic kidney disease, oxidative stress, immune response, complement activation pathway

## Abstract

Chronic kidney disease (CKD) and cardiac insufficiency often co-exist, particularly in uremic patients on hemodialysis (HD). The occurrence of abnormal renal function in patients with cardiac insufficiency is often indicative of a poor prognosis. It has long been established that in patients with cardiac insufficiency, poorer renal function tends to indicate poorer cardiac mechanics, including left atrial reserve strain, left ventricular longitudinal strain, and right ventricular free wall strain (Unger et al., Eur J Heart Fail, 2016, 18(1), 103–12). Similarly, patients with chronic kidney disease, particularly uremic patients on HD, often have cardiovascular complications in addition to abnormal endothelial function with volume overload, persistent inflammatory states, calcium overload, and imbalances in redox responses. Cardiac insufficiency due to uremia is therefore mainly due to multifaceted non-specific pathological changes rather than pure renal insufficiency. Several studies have shown that the risk of adverse cardiovascular events is greatly increased and persistent in all patients treated with HD, especially in those who have just started HD treatment. Inflammation, as an important intersection between CKD and cardiovascular disease, is involved in the development of cardiovascular complications in patients with CKD and is indicative of prognosis (Chan et al., Eur Heart J, 2021, 42(13), 1244–1253). Therefore, only by understanding the mechanisms underlying the sequential development of inflammation in CKD patients and breaking the vicious circle between inflammation-mediated renal and cardiac insufficiency is it possible to improve the prognosis of patients with end-stage renal disease (ESRD). This review highlights the mechanisms of inflammation and the oxidative stress that co-exists with inflammation in uremic patients on dialysis, as well as the mechanisms of cardiovascular complications in the inflammatory state, and provides clinical recommendations for the anti-inflammatory treatment of cardiovascular complications in such patients.

## Introduction

Chronic kidney disease progressing to the uremic stage requires maintenance dialysis therapy to manage complications, prolong patient survival and improve quality of life. Although dialysis treatment is necessary for such patients, the serious cardiovascular complications resulting from hemodialysis (HD) cannot be ignored. HD superimposes additional cardiovascular risks under the cardiac burden of fluid overload due to pre-existing renal disease. The Kidney Disease: Improving Global Outcomes (KDIGO) conference discussed and made clinical recommendations for volume control (Flythe et al., 2020), blood pressure management (Cheung et al., 2021), and pathophysiological changes occurring in the vasculature of CKD patients in the CKD setting (Johansen et al., 2021), and emphasized the necessity of timely hemodialysis for such patients. The guidelines also state that volume overload in patients with CKD leads to cardiac overload in these patients, making them more susceptible to cardiovascular disease, and therefore blood pressure and volume status are important modifiers of clinical outcomes in patients with CKD (Flythe et al., 2015; Zoccali et al., 2017; Assimon et al., 2018). Meanwhile the CKD environment accelerates the progression of central and peripheral arterial disease, especially the onset and progression of atherosclerosis (Johansen et al., 2021).In HD conditions, the microinflammatory state underlying chronic kidney disease can be exacerbated by abnormalities in the immune system, complement activation triggered by blood contact with the dialysis membrane, accumulation of urotoxins and endotoxin translocation, accumulation of inflammatory factors due to decreased renal filtration capacity and local injury due to arteriovenous fistula formation. Inflammation acts as a catalyst for the development of cardiac insufficiency and contributes significantly to the development of cardiovascular complications in these patients. After ischemic myocardium is reperfused by coronary artery, although there is no myocardial necrosis, but the systolic and diastolic depression persists for more than 1 week, and then gradually improves is myocardium stunned (Braunwald and Kloner, 1982; Kloner, 2020). The release of inflammatory mediators directly leads to hemodynamic overload, increases ventricular wall pressure, induces the onset of myocardium stunned, and long-term involvement leads to irreversible myocardial damage Zuidema and Dellsperger, 2012. Repeated circulatory stress leads to myocardium stunned, and the repeated myocardial injury caused by myocardium stunned leads to irreversible changes in the structure and function of the left heart. Also, vascular calcification progresses rapidly in dialysis patients, and vascular calcification, myocardial fibrosis, and reduced left ventricular compliance may make HD patients more susceptible to hypotension and acute pulmonary edema. Impaired autoregulation and the deposition of abnormal substances such as epicardial adipose tissue (EAT) may cause irreversible damage to the myocardium. This paper provides insight into the inflammatory mechanisms underlying the development of cardiac insufficiency in patients with advanced kidney disease on dialysis and provides more systematic theoretical support for clinical interventions for inflammation in these patients.

## Abnormalities of Biomarkers in Dialysis Patients

### Troponin

Increased troponin levels in CKD are attributed to a variety of mechanisms, including increased transmural pressure, small vessel coronary artery obstruction, endothelial dysfunction, intracellular edema, and the direct cytotoxicity of uremia (Arcari et al., 2021). Troponin levels are chronically elevated in patients with renal insufficiency in the absence of myocardial infarction (deFilippi et al., 2012). The pathological mechanisms by which this occurs are inconclusive, but the mechanism of occurrence must be multifactorial and include the development of CKD complications such as hypertension, left ventricular hypertrophy, heart failure, and coronary artery disease (Raber et al., 2021a). Dialysis patients are often monitored for elevated troponin during the dialysis process. This is due to the rapid exchange of fluid through the dialysis membrane, which reduces coronary perfusion and thus induces obstruction or microvascular damage. Troponin is metabolized by the kidneys, which may further elevate the peak troponin levels seen in dialysis-induced coronary artery disease (Chan et al., 2021). This elevated troponin level cannot be explained by ischaemic myocardial necrosis, even if the patient has concomitant coronary artery disease.

Dialysis-induced myocardium stunned also results in elevated troponin, but it is unclear whether the elevated troponin originates from cardiomyocytes with reversible or irreversible damage. What we can determine at this time is that myocardium stunned causes troponin elevation that is not entirely consistent with myocardial ischemic necrosis and that it may be associated with the onset of partial apoptosis.

The highly sensitive cardiac troponin (hs-cTn) assay can detect small troponin concentrations and address small changes in biomarker concentrations (Miller-Hodges et al., 2018; Raber et al., 2021b). However, since the specificity of hs-Tn for monitoring myocardial infarction is reduced in patients with CKD (Twerenbold et al., 2018), we propose to confirm the diagnosis of myocardial infarction by continuous monitoring of troponin and comparing the absolute changes in troponin. This can be interpreted as using the degree of change in troponin as a diagnostic indicator, i.e., it is considered more diagnostic when patients with CKD have a >280% increase in high-sensitivity troponin I (hs-cTnI) or a >250% increase in high-sensitivity troponin T (hs-cTnT), but this also reduces the sensitivity of the diagnosis (Kraus et al., 2018). hs-cTnI and N-terminal precursor B-type brain natriuretic peptide (NT-proBNP) were found to be associated with myocardial fibrosis and myocardial edema by Arcari et al. by comparing cardiac MRI and markers of myocardial injury in patients with different renal functions, and with deterioration. As renal function progressively declined, the serological biomarkers hs-cTnT and NT-pro BNP and imaging markers of structural remodeling correlated more closely with nature T1 (myocardial fibrosis) and T2 (myocardial edema) (Arcari et al., 2021).

### BNPs

hs-cTnI and elevated NT-proBNP levels are associated with myocardial remodeling and its prognosis in patients with chronic kidney disease. Increased markers of myocardial injury in chronic kidney disease may be associated with persistent, non-ischaemic, subclinical myocardial injury (Arcari et al., 2021). Patients with CKD with complications of cardiac insufficiency usually present with preserved left ventricular systolic function and reduced ventricular diastolic function (Ponikowski et al., 20162016; Unger et al., 2016; Arcari et al., 2020).

B-type natriuretic peptide (BNP) and NT-proBNP are elevated due to reduced renal clearance, fluid retention, and abnormal left ventricular function. In particular, NT-proBNP is filtered through the kidneys and regulated by dialysis resulting in large fluctuations in patients with advanced kidney disease, making it impossible to define a threshold value to assess the patient’s cardiac function (Chan et al., 2021).

### sST2

soluble suppression of tumorigenicity (sST2) has received much attention in recent years as a new biomarker for risk stratification in acute and chronic heart failure, for therapeutic assessment, and for predicting patient prognosis (Gaggin and Januzzi, 2013; Januzzi et al., 2015; Dalal et al., 2018; Mirna et al., 2020). A 2018 clinical study assessing the relationship between sST2 and renal function in 842 patients with CKD noted that sST2 was associated with progressive renal function, with higher sST2 suggesting lower estimated glomerular filtration rate (eGFR) (Alam et al., 2019). Subsequently, Mirna et al. ^21^studied five new biomarkers in 219 patients with CKD and found that all investigated biomarkers were significantly elevated in patients with CKD, inversely related to eGFR, except for sST2. sST2, as one of the biomarkers with the least impact on changes in renal function, could act independently of renal function. Therefore, sST2 is of great importance for clinical practice in CVD patients with combined CKD.

### suPAR

The urokinase plasminogen activator receptor (uPAR) is a binding receptor expressed on the surface of immune cells (mainly neutrophils, activated T cells, and macrophages). Stimulation by inflammation causes cleavage of the uPAR on the cell surface, followed by the production of soluble uPAR, Soluble urokinase plasminogen activator receptor (suPAR) (Huai et al., 2006; Allison, 2016). Thus, elevated suPAR reflects inflammation and immune system activation and is an emerging marker of inflammation (Huttunen et al., 2011).

In 2010, Eugen-Olsen et al. evaluated the correlation between suPAR levels and the risk of cancer and CVD in 2602 general population and suggested that suPAR could be an independent risk factor for predicting the incidence of CVD and all-cause mortality (Eugen-Olsen et al., 2010). In 2014 Borné et al. followed 4,530 general population for a median time of 16.3 years and found that suPAR was associated with elevated NT-proBNP plasma levels and incidence of HF (Borné et al., 2014). Subsequently, Hodges et al. demonstrated that suPAR can outperform traditional inflammatory markers, such as C-reactive protein (CRP), as a biomarker for cardiovascular disease in the prediction of the risk of CVD development (Hodges et al., 2015).

In the kidney, suPAR can induce podocyte dysfunction and impair glomerular filtration function thus leading to the development of CKD (Hayek et al., 2015; Lv et al., 2020), a phenomenon mostly found in focal segmental glomerulosclerosis studies (Wei et al., 2011). 2019 a meta-analysis of the relationship between suPAR and kidney disease pointed out that suPAR was negatively correlated with eGFR, and in patients with CKD, especially ESRD patients suPAR levels were significantly higher than normal (Shuai et al., 2019). Several subsequent studies suggested that close monitoring of suPAR could help in the early diagnosis and treatment of CKD and that suPAR levels were associated with CKD prognosis (Rotbain Curovic et al., 2019; Shuai et al., 2019). 2021 Jhee et al. evaluated the relationship between disease progression and suPAR in 751 CKD patients and demonstrated that suPAR levels were also independently associated with CKD progression (Jhee et al., 2021).

## Inflammation

In its normal state, inflammation has a protective effect on infected and damaged tissues by dilating blood vessels and recruiting white blood cells and plasma proteins to abnormal tissues. However, when inflammation persists and is poorly controlled, it can lead to a range of complications. The pathophysiology of chronic inflammation in CKD is not fully understood, but the prognosis of such patients is closely related to their inflammation *in vivo*. The development of inflammation in ESRD patients is multifactorially induced, including 1) exposure of blood to exogenous substances such as dialysis membranes during HD that stimulate inflammation and activate the complement pathway; 2) immune dysfunction, including senescence and apoptosis of immune cells; 3) accumulation of urotoxins *in vivo*, secondary to intestinal dysregulation; and 4) unbalanced oxidative stress (Sun et al., 2016), all of which are discussed later. In addition, increased circulatory preload due to reduced renal function and the presence of metabolic acidosis can lead to increased production of pro-inflammatory factors. Therefore, many inflammatory factors (CRP, Interleukins, etc.) predict the prognosis of ESRD patients by effectively assessing the degree of inflammation in such patients.

### Coexistence of Immunity With Inflammation and Ongoing Immune Stimulation

The persistence of immune abnormalities in ESRD patients is a dominant factor in the development and progression of inflammation and mediates inflammation in conjunction with reduced renal clearance due to deteriorating renal function (Stenvinkel et al., 2005). Among these, neutrophil- and monocyte-mediated innate immune responses and lymphocyte-mediated adaptive immune responses dominate the immune abnormalities in ESRD patients. In addition to this, patients on long-term maintenance dialysis (MHD) are also characterized by normal immunoglobulin levels, disrupted antigen-presenting cells (APCs), and upregulation of phagocyte supramastigote receptors (SRs) (Kato et al., 2008).

#### Innate Immune Response: Neutrophils and Monocytes

Neutrophils and monocytes, as the most important cells in the immune response to hemodialysis-mediated urotoxicosis and long-term contact with biologically incompatible membranes, coordinate the immune response through the production of cytokines and chemokines by recognizing pathogens or damaged tissues together with dendritic cells and natural killer (NK) cell (Losappio et al., 2020). Therefore, changes in the number of neutrophils and NK cells and changes in phagocytic activity in HD patients may indicate abnormalities in the body’s immune system, and such abnormalities are related to the type of biologically incompatible membrane and the dialysis method (Colì et al., 1999; Moore et al., 2001; Pappas et al., 2019). Premature cellular senescence in ESRD patients is caused by persistent DNA damage and epigenetic changes, usually in the form of cellular cessation of proliferation and apoptosis (Kooman et al., 2017; Crépin et al., 2020). This is accompanied by an excessive accumulation of senescent polymorphonuclear neutrophils due to neutrophil dysfunction (Martin et al., 2003). Neutrophils are important cells of the innate immune system and in the early stages of HD treatment, there is a transient decrease in neutrophils due to apoptosis. Anti-myeloperoxidase antibodies (MPO), a type of anti-neutrophil cytoplasmic antibody (ANCA), are a functional marker and activation marker of neutrophils, and changes in their levels and activity are representative of the functional and active status of neutrophilic polymorphonuclear leukocytes (PMN). The excessive apoptosis of neutrophils due to increased MPO release is thought to be the pathological mechanism underlying the development of microinflammation in HD patients (Fukushi et al., 2020), leading to an increased risk of infection and mortality from infection in patients starting HD. This mechanism also confirms the conclusion that plasma MPO is an independent risk factor for all-cause mortality in HD patients, as suggested by two clinical studies in recent years (Nakayama et al., 2018; Kim et al., 2020). Early studies attributed the decrease in neutrophils to their accumulation in the capillaries of the lungs. Recent studies have suggested that the contact of blood with the dialysis membrane leads to the recruitment and activation of neutrophils and monocytes, and that activated neutrophils attach to the endothelial wall of the lung capillaries, the first vascular surface they come into contact with after leaving the dialyzer, resulting in a transient decrease in neutrophils (Hoenich et al., 1986). Transient leukopenia is caused by the activation of the alternative pathway (AP) and the lectin pathway (LP) of complement after the blood comes into contact with the dialysis membrane (Yoon et al., 2007). Further immune dysfunction will result in the release of pro-inflammatory cytokines [e.g., interleukin (IL)-1β, IL-6, IL-8, tumor necrosis factor-alpha (TNF-α), monocyte chelator protein-1 (MCP-1), and gamma interferon (Hempel et al., 2017)] from activated neutrophils and monocytes, along with activation of the complement system (dell'Oglio et al., 2017). Activation of the complement system leads to increased expression of adhesion molecules [i.e., CD11b/CD18, also known as complement receptor 3 on leukocytes (CR3)]*in vivo*, which in turn binds to C3b on the dialysis membrane and further leads to neutropenia. The adhesion factors themselves can also induce leukocyte extravasation. CR3 can interact with platelets to cause thrombosis and release factors that stimulate thrombosis (e.g., Von Willebrand factor) (Losappio et al., 2020).

#### Adaptive Immune Response: Lymphocytes

Patients with ESRD have an abnormal body environment resulting in a chronic inflammatory state characterized by increased production of pro-inflammatory cytokines by T cells, high levels of circulating follicular helper T cells (T_FH_), and abnormal maturation of plasma cells and T helper (Th) lymphocytes (Losappio et al., 2020). High levels of cytokines are present in ESRD patients due to abnormal renal filtration and a persistent inflammatory state. Notably, IL-18 leads to the development of T cell-mediated adaptive immune changes by inducing the onset of Th1-mediated immune responses and activating Th2 immune responses through the production of IL-4 and IL-13 (Mühl et al., 1996). At the same time in ESKD patients, prolonged inflammatory signaling alters T-cell function and leads to T-cell failure (Wherry and Kurachi, 2015). In a sustained state of inflammation, the abnormal T-cell function changes are irreversible and eventually lead to cell death. A significant reduction in lymphocytes has been identified in several clinical studies of CKD, and the remaining T lymphocytes in such patients may exhibit a more sustained and active pro-inflammatory state (Hartzell et al., 2020). As a subset of CD4^+^ T cells, T_FH_ helps B cells to produce high-affinity antibodies against pathogens that are potentially pathogenic in chronic inflammatory states (e.g., atherosclerosis, lymphoid tumors, autoimmune diseases) (Crotty, 2019; Hartzell et al., 2020). In addition, the production of IL-4 by T_FH_ may also contribute to the differentiation of macrophages, which play an important pro-inflammatory role in ESRD patients, towards a subset of macrophages that are more capable of producing cytokines and chemokines (Guiteras et al., 2016).

During HD treatment, the body’s immune response is weakened due to a significant reduction in levels of B-cell activating factor (BAFF) and IL-17 receptors, followed by a significant reduction in B-lymphocytes. At the same time, the expression of the B-cell lymphoma-2 gene (Bcl-2, an oncogene that significantly inhibits apoptosis) decreases B lymphocytes that are more susceptible to apoptosis. In addition, CD40, a functionally relevant surface antigen for T and B lymphocytes, is essential for B cell growth and primarily promotes the proliferation of immature B cells (Ferraccioli and Gremese, 2017). In patients with CKD, particularly those receiving HD, serum levels of CD40 are elevated (Esposito et al., 2012). Clinical interventions for CD40, such as filtration of CD40 by dialysis membranes may therefore provide additional benefit to such patients.

#### Neutrophil-Lymphocyte Ratio

The neutrophil-lymphocyte ratio (NLR), the most sensitive and specific of the inflammatory biomarkers, is an effective indicator of inflammatory status in patients with ESRD (Ahbap et al., 2016). 2 clinical studies published in 2020 showed that NLR is a predictor of all-cause mortality and cardiovascular mortality in patients with chronic kidney disease and that higher NLR indicates higher mortality, so early clinical intervention is indicated in patients with high NLR and CKD (Zhao et al., 2020; Ao et al., 2021). The QRS-T angle is considered to be a response indicator for myocardial inhomogeneity and is the strongest predictor of cardiac death (Yamazaki et al., 2005). Boltuc et al. compared the difference in QRS-T angle between patients with advanced kidney disease on hemodialysis and those with normal eGFR and concluded that the QRS-T angle was significantly increased in HD patients and that NLR was strongly associated with all-cause and cardiac death were strongly associated (Boltuc et al., 2020). Several studies have shown that NLR has a higher sensitivity and a stronger cardiovascular prognostic association than CRP. Thus, NLR may provide additional clinical benefits to HD patients as a new indicator of inflammation.

Weakened immune function in patients with CKD, particularly in MHD, exacerbates pre-existing infections and activates immunity, which in turn leads to inflammation. Alterations in the body’s immune cells accelerate atherosclerosis (Fernandez et al., 2019) and weaken the body’s ability to clear pathogens and tumor cells, leading to a significantly increased risk of cardiovascular death, infection, and malignancy in patients with renal failure (George et al., 2017). Immune dysfunction is, therefore, an important reason for the reduced survival years of ESRD patients.

### Blood Contact With Dialysis Membranes Activates the Complement System

Craddock et al. were the first to find a predisposition to acute cardiopulmonary insufficiency in the early stages of HD treatment. This is since in patients newly receiving HD, blood contact with dialysis membranes can stimulate innate immune activation and the body recognizes the exposed biological material as a non-self antigen that promotes inflammation through immune cell stimulation, inflammatory cell aggregation, complement activation, and cytokine production (Table 1) (Honkanen et al., 1991; Pertosa et al., 1993; Rousseau et al., 1999). Complement is one of the major components of the innate immune system and bridges the adaptive response of the body to abnormal stimuli. c3 acts as a sink for the complement activation pathway and plays a pivotal role in the activation of the complement system and is a key molecule in the activation of the alternative pathway. c5a acts directly on vascular endothelial cells to increase vascular permeability. It is also a chemotactic agent for neutrophils and monocytes at high concentrations, driving the directional movement of these cells, stimulating the oxidative metabolism of neutrophils and monocytes, stimulating neutrophil adhesion, and even increasing oxidative stress (Simone et al., 2014). In addition, C5a enhances the immune response and induces the secretion of cytokines such as IL-1, IL-6, IL-8, and TNF-α by monocytes, which promotes the proliferation of T cells and antibody production by B cells. Meanwhile, increased production of C3a and C5a and lecithin (C3b, iC3b) in HD patients leads to increased cytokine production, exacerbates cytotoxicity, and is mediated by the release of granzyme from neutrophils, promoting inflammation. However, we believe that this complement activation effect is only active in the early stages of HD and gradually decreases during long-term dialysis, and some studies have confirmed a negative correlation between C3 levels and dialysis duration (Girndt et al., 1999; Buraczynska et al., 2009; Mares et al., 2009; Mares et al., 2010; Inoshita et al., 2012; Kishida et al., 2013; Hornum et al., 2014; Poppelaars et al., 2018).

**TABLE 1:** Complement is activated by three pathways: the alternative pathway (AP), the lectin pathway (LP), and the classical pathway (CP). Complement activation can be induced by adsorption of complement components to hemodialysis membranes, with AP and LP being the major activation pathways.AP activity is increased by spontaneous C3 hydrolysis that continuously applies minor stimuli to AP.C3b generated by C3 hydrolysis also enhances CP and LP.Under dialysis conditions, covalent binding of C3b to nucleophilic surfaces expressed on dialyzer membranes and adsorption of CHF by dialysis membranes promote activation of the complement substitution pathway, and complement factor B acts as an intermediate mediator involved in the continuous occurrence of complement activation (Flythe et al., 2015). LP is induced by the binding of MBL or Ficolin to carbohydrates and is activated by the adsorption of large amounts of Ficolin-2 and MBL on dialysis membranes during HD, and a significant decrease in Ficolin-2 levels can be found in these patients. CP is induced by the binding of C1q to molecules such as immune complexes or CRP and is activated in HD patients mainly by C1q binding to In HD patients, it is mainly activated by C1q binding to circulating IgG.

Complement activation pathway	Mechanism of occurrence under physiological conditions	Mechanism of complement activation occurring in hemodialysis	Mediating the development of cardiovascular disease
AP	It continuously stimulates complement activation through spontaneous C3 hydrolysis and enhances CP and LP through C3b production	Reduced expression of complement inhibitors leads to AP dysregulation: polysulfone dialyzers can absorb ^c^CHF (an important inhibitor of C3 convertase and C3b) and clusterin (blocks activation of the terminal pathway) (Unger et al., 2016)	The Y402H genotype in CFH increases the risk of cardiovascular disease in HD patients (Chan et al., 2021)
LP	^a^MBL or Ficolins recognize carbohydrate-induced	^b^Ficolins-2 initiates complement cascade reactions (including C5a production) and dialysis-induced leukopenia by adsorption to polysulfone dialyzers and leads to substantial depletion of Ficolins2. MBL also activates complement reactions by contact with dialyzers (Unger et al., 2016; Flythe et al., 2020)	MBL is involved in the consumption of atherogenic particles and is beneficial for atherosclerosis in uremic patients. Thus a decrease in MBL (adsorbed to the dialyzer) in HD patients is associated with an increased risk of cardiovascular disease (Cheung et al., 2021)
			C5a is involved in thrombosis. activation of the LP pathway is significantly associated with increased production of C5a, especially during the first hour of HD onset (Johansen et al., 2021)
CP	Induced by C1q binding to immune complexes or other molecules (e.g., CRP)	C1q binds to immunoglobulin IgG adsorbed by the membrane dialyzer to activate the complement response	C1q, the largest molecular weight gamma globulin among complement components, can promote the release of inflammatory mediators from eosinophils and mast cells under HD conditions, leading to vascular endothelial damage and subsequently atherosclerosis (Zoccali et al., 2017)

a MBL, mannose-binding lectin;

b Ficolins-2: specific pathogen recognition receptor for LP that acts similarly to MBL;

c CHF, complement factor H, inhibits C3 convertase and C3b activity while acting negatively on alternative pathways.

**FIGURE 1:**
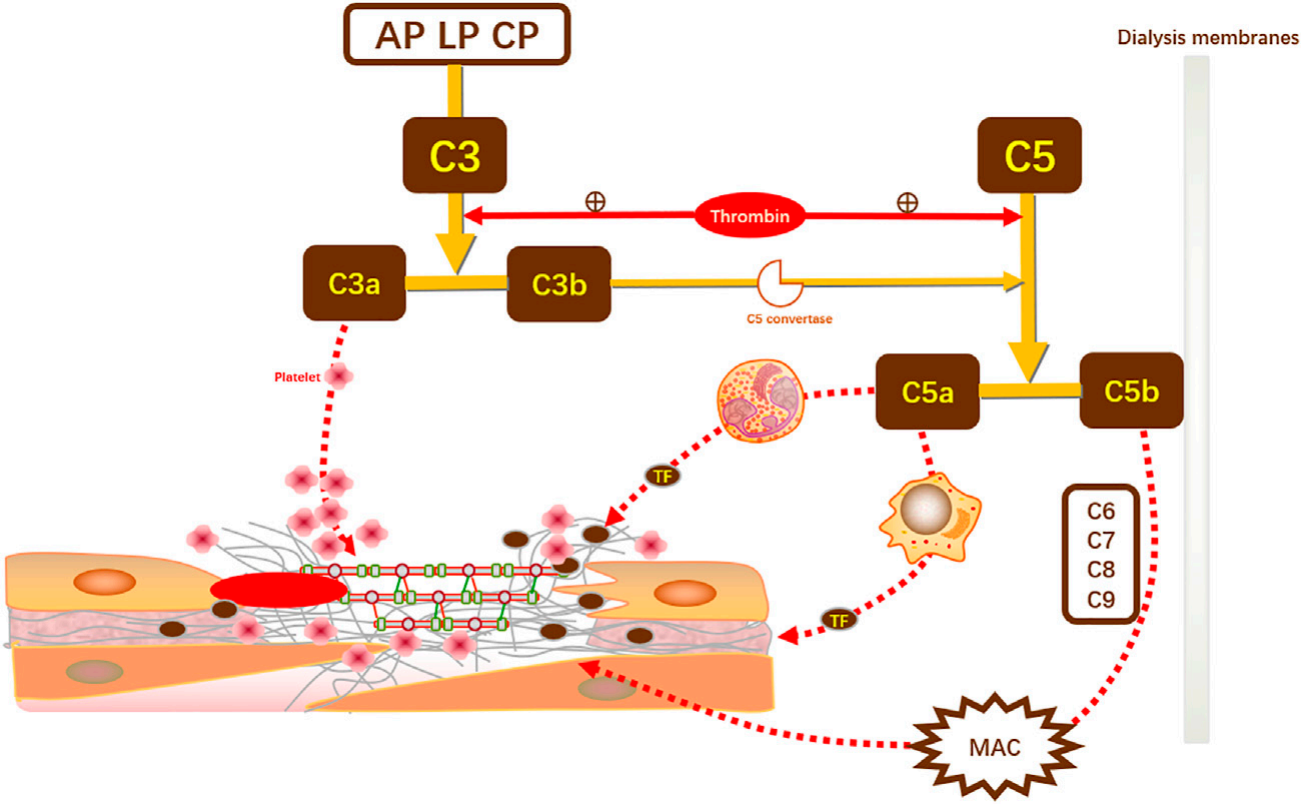
Complement activation promotes coagulation. C3, the initiator of the complement activation pathway, can be cleaved into effector components, namely C3a and C3b. In CKD-induced complement activation, C3a directly promotes coagulation by enhancing platelet aggregation and adhesion. Meanwhile, C3b promotes the synthesis of C5 convertase to induce C5 cleavage to produce C5a and C5b. C5a directly stimulates neutrophils and monocytes to increase the expression of TF and thus induce thrombosis. In renal replacement therapy, C5b comes into contact with the dialysis membrane and, together with multiple complements (C6–C9), mediates the production of MAC and induces coagulation. In turn, coagulation secondary to complement activation can amplify complement and coagulation activation through positive feedback from thrombin on C3 cleavage. TF, tissue factor; MAC, membrane attack complex; AP, alternative pathway; LP, lectin pathway; CP, classical pathway.

The presence of a procoagulant state during HD due to complement activation increases the risk of inflammatory and cardiovascular events and may result in a poor prognosis (Poppelaars et al., 2018). Complement effectors have a procoagulant effect. First, C3a can activate platelet aggregation and adhesion. Second, C5a can stimulate the expression of tissue factor and tissue-type fibrinogen activator inhibitors *in vivo* by centrophils and monocytes to promote thrombus formation. In turn, the coagulation component thrombin can cleave C3 into C3a and C3b and C5 into C5a and C5b, thereby amplifying the activation of complement. For the treatment of coronary vascular lesions presenting on dialysis, invasive therapy has not been beneficial in most studies due to the risk of contrast nephropathy occurring, and Bangalore et al. even suggested that invasive therapy may increase the risk of stroke in patients with advanced CKD (Figure 1). Not only that, with coronary revascularization, but reperfusion to the myocardium may also lead to more severe myocardial injury, i.e., myocardial ischemia-reperfusion injury, due to mechanisms such as oxidative stress, calcium overload, apoptosis, and leukocyte accumulation (Bangalore et al., 2020).

### Uremic Toxin Involvement With Endotoxemia

The development of endotoxemia and the accumulation of uremic toxins contribute to the specific pattern of inflammation in ESRD patients (Crépin et al., 2020). Bacterial endotoxin is a lipopolysaccharide of the outer membrane of Gram-negative rods, which accounts for 70% of the total intestinal bacteria in the healthy population. Endotoxin is broken down by the bacterial cell wall inside and outside the intestinal lumen and is released through host defense and self-integration mechanisms. It has a strong stimulatory effect on the development of inflammation, increasing pro-inflammatory cytokine release by stimulating monocytes/macrophages and circulating lipopolysaccharide receptors to bind to systemic immunoreceptor cells (Yang et al., 1998). Endotoxin translocation by crossing the intestinal barrier into the circulation, i.e., endotoxemia, occurs, which is dependent on abnormal intestinal permeability, and this occurs mainly in cases of intestinal edema and intestinal hypoperfusion. Endotoxemia is associated with systemic inflammation, oxidative stress, malnutrition, cardiac injury, and poor prognosis in cardiovascular disease (Krack et al., 2005; Kotanko et al., 2006).

Uremic toxins are substances that are significantly elevated in patients with renal failure that are toxic, such as urea, creatinine, peptides, potassium ions, and indole sulfate (IS). Gastrointestinal stasis due to excess fluid and reduced clearance of uremic toxins in patients with renal insufficiency results in altered gastrointestinal permeability and loss of intestinal epithelial barrier integrity resulting in exposure to significant endotoxemia, which is characteristic of every patient with CKD (Rysz et al., 2021a; Rysz et al., 2021b). When dialysis is started in CKD patients, the body undergoes significant hemodynamic disturbances, and HD-induced systemic circulatory stress and repeated local ischemia of important organs (here mainly the mesentery) lead to damage of the intestinal mucosa, which subsequently leads to increased translocation of intestinal endotoxins. In normal subjects, endotoxins in the intestine enter the liver through the portal vein and are then removed (Yu et al., 1997; McIntyre et al., 2011). Hemodialysis alters hepatic blood flow and diminishes liver function, leading to endotoxemia and thus increased inflammation (Marants et al., 2021). As a major structural component of the outer membrane of the cell wall of Gram-negative bacteria, the shift of LPS to the inflammatory isoform and the decrease in LPS function is strongly associated with the development of severe inflammation in ESRD patients. Regulation of the gut microbiota may prevent the development of endotoxin-induced inflammation in such patients (Adda-Rezig et al., 2021).

At the same time, uremic toxins mediate abnormal endothelial function in CKD patients. is directly contributes to cardiovascular complications by reducing NO synthesis in endothelial cells and successively impairing endothelial cell proliferation is also indirectly contributes to cardiotoxicity through pro-inflammatory effects and altered immune processes (Düsing et al., 2021; Rapa et al., 2021). Uremic toxin as an intermediate mediator of cardiac and renal damage reflects the complex relationship between heart and kidney, highlighting a potential target for prevention of cardiovascular complications in CKD patients.

Endotoxemia is associated with the occurrence of cardiovascular events in HD patients, highlighting the potential toxicity inherent in HD and providing a clearer therapeutic strategy for improved clinical management.

### Hemodialysis Access

Hemodialysis access is usually performed using either an autologous arteriovenous fistula (AVF) or a central venous catheter (CVC). Among them, AVF is the first choice of arteriovenous access for hemodialysis, which can ensure the adequacy of dialysis and maximize the prognosis of patients (Ethier et al., 2008). The risk of sepsis in HD patients is significantly associated with hemodialysis access and is associated with a dramatic increase in mortality (Locham et al., 2021). 2021 Valtuille et al. noted that patients on dialysis with AVF had less oxidative stress or lower severity of oxidative stress relative to patients on long-term central venous catheter dialysis (CVC) (Valtuille et al., 2021). In maintenance hemodialysis (MHD) patients, AVF has a protective effect against unbalanced peroxidation-oxidation. In contrast, patients on dialysis with CVC had a more pronounced inflammatory state (e.g., elevated CRP and IL-6) and higher mortality (Banerjee et al., 2014; Chan et al., 2019). Nevertheless, AVF induces inflammation in HD patients, firstly by stimulating the production of oxidative stress markers during endovascular fistula formation, inducing intimal hyperplasia, and exacerbating local inflammation. Second, AVF for hemodialysis can lead to a hyperdynamic state of circulating blood, stimulating left ventricular hypertrophy and leading to a poor cardiac prognosis (Lee et al., 2021).

### High Levels of Inflammatory Mediators

The kidney, as an important organ, receives 1/4 of the total blood flow. Under conditions of impaired renal function, the kidney becomes a target of persistent chronic inflammation due to impaired antioxidant and anti-inflammatory defenses and detoxification. In turn, due to increased inflammatory factor production and impaired proximal tubular clearance, blood concentrations of acute-phase proteins (CRP and fibrinogen) and pro-inflammatory cytokines (IL-1β, IL-6, and TNF-α) increase progressively as renal function deteriorates (Gupta et al., 2012; Hartzell et al., 2020). Pro-inflammatory cytokines alter the adhesion molecules on the surface of endothelial cells and leukocytes in the renal vasculature and disrupt the glycocalyx layer (a negatively charged villi-like structure covering the vascular endothelium that is involved in regulating vascular permeability, regulating leukocyte adhesion and flow, responding to vascular mechanical shear, and inhibiting intravascular coagulation), leading to changes in endothelial barrier function, vascular reactivity, and the coagulation system, and even disrupting renal physiology. This alteration also explains the mechanism of cardiovascular complications in CKD patients (Gupta et al., 2012; Hartzell et al., 2020).

Although a small study in 2021 noted that long-term maintenance hemodialysis reduces blood levels of inflammatory factors (including IL-2, IL-6), and high-sensitivity C-reactive protein (hs-CRP)] in patients with renal failure, inflammation remains the dominant factor for cardiac insufficiency that occurs at the start of dialysis (Zhou et al., 2021). In 2016 Sun et al. first compared multiple biomarkers in patients with advanced kidney disease on hemodialysis, and the study suggested that most inflammation-related biomarkers are elevated in such patients, and all of them play a predictive role in the occurrence and prognosis of cardiovascular events in such patients (Mun and Golper, 2000; Sun et al., 2016). There is a correlation between most biomarkers, led by IL-6 and hs-CRP. Multiple biomarkers are collectively involved in the occurrence of cardiovascular events in patients with advanced kidney disease, and the degree of change in biomarkers reflects some extent the possible concurrent diseases in patients with chronic kidney disease, suggesting a general alteration in the signaling pathways of inflammation and apoptosis in uremic and dialysis patients with the uremic disease (Sun et al., 2016). We listed the inflammatory mediators that are closely associated with inflammation and cardiovascular disease development in HD patients and explored the adaptive changes that occur in the organism (Table 2).

**TABLE 2:** A recent study of common inflammatory factors associated with cardiovascular complications in hemodialysis patients.

Cytokines	Clinical studies related to cardiovascular complications
IL-1	Several animal studies have shown additional clinical benefits to the kidney with IL-1β inhibitors (Assimon et al., 2018)
	A 2017 randomized, double-blind, placebo-controlled trial of more than 10,000 patients with CKD by Ridker et al. showed that the use of a human monoclonal antibody targeting IL-1β was associated with a significant reduction in the recurrence of cardiovascular events in such patients (Kloner, 2020)
IL-6	In a 5-year follow-up study of 45 patients on long-term hemodialysis, Thang et al. demonstrated that IL-6) had a more powerful predictive prognostic significance for cardiovascular disease than CRP in HD patients (Braunwald and Kloner, 1982)
	This was corroborated in a 2015 case study of multiple biomarker levels in 543 ESRD patients, confirming that IL-6 is a strong independent predictor of clinical outcome in patients with CKD (Zuidema and Dellsperger, 2012; Arcari et al., 2021)
	A case study in 2021 evaluated the genetic phenotypic differences in IL-6 and its predictive value for all-cause mortality in 289 ESRD patients and found that the IL6 (−174G > C) (r1800795) polymorphism regulates the inflammatory response in ESRD patients. The CC genotype, a less common IL6 genotype, causes more severe inflammation and suggests a poorer prognosis in ESRD patients (deFilippi et al., 2012)
IL-18	A 2015 study of the prognosis of patients with CKD who had an acute myocardial infarction 1 year earlier concluded that IL18 was a significant predictor of cardiogenic death at 2-year follow-up (Raber et al., 2021a)
CRP	A 2021 study assessing the correlation between dialysis adequacy and inflammation in 536 HD patients using CRP as an indicator of inflammation noted that inadequate dialysis doses may lead to higher levels of inflammation in chronic hemodialysis patients. And high levels of CRP were directly correlated with neutrophil-lymphocyte ratio and serum albumin (Raber et al., 2021b)
sAlb	The relationship between changes in albumin and sAlb and prognosis in patients transitioning from CKD to ESRD stage was studied for the first time in 2019. Patients just transitioning from CKD to ESRD have a high short-term mortality rate, so improving the nutritional status of pre-ESRD patients, including sAlb levels, is important for the prognosis of such patients (Miller-Hodges et al., 2018). lower pre-ESRD sAlb is significantly associated with higher all-cause, cardiovascular and infection-related mortality and hospitalization rates after ESRD (Twerenbold et al., 2018)
	In 2020 Amanda et al. compared sAlb and its prognosis across renal function and found that despite the apparent correlation between sAlb and eGFR, a significant correlation between sAlb and mortality in patients with CKD was observed in multiple subgroups classified by renal function (Kraus et al., 2018). It was also noted that sAlb <4.6 g/dl suggested higher mortality and that maintaining sAlb between 4.6 and 4.8 g/dl may be more beneficial in CKD patients with eGFR <60 ml/min/1.73 m^2^. Therefore, we suggest that dietary protein intake should be moderate for patients with CKD and that high sAlb should not be the therapeutic goal (Ponikowski et al., 20162016; Arcari et al., 2020)

#### IL-1

Interleukin 1 (IL-1), also known as a lymphocyte-stimulating factor, is produced by activated monocytes-macrophages. The physiological functions of IL-1 include 1) stimulating the activation of T lymphocytes, 2) stimulating B cell proliferation and antibody secretion, 3) enhancing NK cell activity in concert with IL-2 or interferon, and 4) inducing the release of inflammatory mediators from neutrophils, thus participating in immune regulation. Due to the specific role of IL-1 on the immune system, therapy to inhibit IL-1 function has emerged as a potential therapeutic target to weaken inflammation in CKD patients, especially in HD patients (Barreto et al., 2010; Ridker et al., 2018; Düsing et al., 2021). Inhibition of IL-1β offers a broader prospect for the treatment and prevention of cardiovascular complications in CKD patients (Ridker et al., 2018).

#### IL-6

Interleukin 6 (IL-6) is an intermediate mediator of the acute response and enhances defense mechanisms against external stimuli by triggering the body’s alarm signals through timely expression. IL-6 can stimulate inflammatory and autoimmune processes by stimulating acute phase protein synthesis and neutrophil production (Babaei et al., 2014). IL-6 concentrations are high in CKD patients, especially in maintenance hemodialysis patients, due to the accumulation of uremic toxins, failure of renal filtration capacity, volume overload, and stimulation by oxidative stress (Babaei et al., 2014; Cao et al., 2014). Likewise, the dialysis process helps stimulate an inflammatory response that further increases IL-6 production.

IL-6 is the only independent predictor of the risk of cardiovascular comorbidity, cardiac mortality, and all-cause mortality in patients with CKD, better reflects the relationship between inflammation and cardiovascular complications than other cytokines (Kisic et al., 2016) and is the most reliable predictor of cardiovascular mortality and total mortality found in patients with CKD (Barreto et al., 2010; Sun et al., 2016).

#### IL-18

Interleukin 18 (IL-18) acts as a potent pro-inflammatory cytokine, triggering the production of a series of pro-inflammatory cytokines by producing immediate inflammatory factors (i.e., TNF-α and IL-1β), among others.

IL-18 is involved in the development of atherosclerosis and contributes to plaque instability through an immune response that increases the synthesis of endothelial adhesion molecules by inducing the synthesis of interferon-gamma (IFN-γ) and reduces the size of the fibrous plaque cap by promoting the expression of major histocompatibility complex II (MHC II) on macrophages and vascular cells. On this basis, IL-18 regulated matrix metalloproteinase upward expression thereby inhibiting collagen synthesis. Combining several clinical studies, we propose that IL-18 not only accelerates the progression of atherosclerosis in CKD patients but also has a predictive value for cardiovascular prognosis in CKD patients (Sasaki et al., 2021).

In addition, IL-18 is involved in the overall progression of inflammation by mediating the production and activation of various cytokines. in addition to the aforementioned alteration of the Th1/Th2 ratio leading to an altered T-cell adaptive response (Mühl et al., 1996), IL-18 can also synergistically activate Th17 cells with IL-23 to promote the production of the cytokine IL-17 and thus induce the recruitment of neutrophils and monocytes to the site of inflammation (Poulianiti et al., 2016).

#### C-Reactive Protein

Several previous studies have shown high levels of CRP in patients with intermediate to advanced CKD, with higher levels in ESRD and dialysis patients (Spoto et al., 2015).

It was found that CRP >3 mg/L in dialysis patients may indicate the occurrence of adverse events and a doubling of the risk of cardiovascular disease but has no significant effect on all-cause mortality. Inflammation plays a more important role than oxidative stress in the mechanism of cardiovascular disease in HD patients (Sasaki et al., 2021).

#### Serum Albumin

Serum albumin (sAlb) is an important biomarker synthesized by the liver and its production is associated with the nutritional status of the body and inflammation (Kim et al., 2013). Patients with CKD often have low sAlb levels, which are associated with lower quality of life and higher rates of hospitalization and mortality due to the imbalance in serum albumin synthesis and consumption caused by the chronic inflammatory state and the susceptibility to other co-morbidities (Friedman and Fadem, 2010; Kato et al., 2010). Serum albumin levels are determined by the rate of hepatic synthesis and catabolism, protein intake, and blood volume distribution, so changes in its concentration can assess the treatment outcome and prognosis of patients receiving renal replacement therapy (Herselman et al., 2010). Not only that, but CKD patients with hypoalbuminemia who develop ESRD are more likely to develop comorbidities such as diabetes mellitus and congestive heart failure (Hsiung et al., 2019). Inflammation interacts with sAlb. Protein-energy wastage (PEW) is often seen in patients with CKD and is an important reason for the poor prognosis of such patients (especially hemodialysis patients). When the body is in a situation of protein and energy depletion, it stimulates inflammation, which in turn leads to PEW, the main indicator of PEW, namely serum albumin (Kovesdy and Kalantar-Zadeh, 2009). On the other hand, lower sAlb is associated with higher validation markers (CRP and leukocytes/neutrophils) (Hsiung et al., 2019). The increased concentration of uremic toxins that occurs due to dietary restrictions in CKD patients leads to altered gut microbiology in such patients stimulating inflammation and a decrease in sAlb (Rothschild et al., 1973). Dietary restrictions in patients with CKD lead to alterations in the gut microbiology of such patients, and the resulting increase in uremic toxin concentrations that occur stimulates inflammation and a decrease in sAlb (Pecoits-Filho et al., 2002; Johansen et al., 2010; Shahzad et al., 2015; Ridker et al., 2017; Thang et al., 2020). In addition, sAlb also decreases due to reduced physical activity in HD patients. In patients on maintenance hemodialysis, the dynamics of serum albumin concentration is an independent predictor of all-cause mortality and cardiac mortality, and in 2010 it was shown that lower serum albumin predicted higher all-cause mortality and cardiovascular mortality (Herselman et al., 2010).

## Oxidative Stress Mechanisms Parallel to Inflammation

Oxidative stress (OS), which refers to an imbalance between the two antagonistic systems of oxidation and antioxidation in the body, tends to oxidize, causing abnormalities in the biochemical and physiological processes of the body and damage to endothelial tissues (Formanowicz et al., 2015; Kalantar-Zadeh et al., 2016; Kalantar-Zadeh et al., 2017; Ko et al., 2017; Brown-Tortorici et al., 2020; Rocha et al., 2021; Valga et al., 2021). Oxidants of oxidative stress refer to reactive oxygen or nitrogen species (ROS or RNS, respectively) as well as free radicals. Centrophages and monocytes/macrophages are the main sources of ROS, and oxidative stress increases the production of chemokines (MCP-1, CSF-1) and adhesion molecules (ICAM-1), tending the redox balance towards a peroxidized state by promoting the aggregation of these cells (Vaziri et al., 2003). The constant pathological stimulation of macrophages in the inflammatory state can promote oxidative stress and lead to excessive production of ROS, while ROS-induced activation of transcription factors and pro-inflammatory genes can in turn increase inflammation (Chu et al., 2020; Podkowinska and Formanowicz, 2020).

Oxidative stress in chronic kidney disease is mediated by multiple factors, mainly due to impaired antioxidant mechanisms and increased production of reactive oxygen species (ROS) (Düsing et al., 2021). To achieve redox homeostasis under physiological conditions, highly active antioxidant mediators [including catalase (CAT), glutathione peroxidase (GPX), and the free radical scavenger superoxide dismutase (SOD)] are present in the glomerulus (Siems et al., 2002; Jagiela et al., 2020). The excessive production of ROS negatively affects various components of the renal unit, impairing the glomerular microcirculation and leading to glomerular ischemia in the long term (Luczak et al., 2011). Oxidative stress leads to apoptosis and necrosis of tubular epithelial cells, increased synthesis of collagen and fibronectin by endothelial and thylakoid cells, leading to tubular atrophy and interstitial fibrosis. With the loss of renal function and destruction of a tubular structure, renal regulatory mechanisms, such as the Renin-Angiotensin-Aldosterone system (RAAS), are affected, rendering the kidney unable to compensate for electrolyte and acid-base homeostasis disturbances. OS is more severe in HD patients, and investigators have attributed the oxidative stress state in HD patients to 4 main causes, including urotoxicosis, dialyzer interaction, dialysate contamination, and peripheral blood cell-dialysis membrane interaction (Valtuille et al., 2021).

Reactive oxygen metabolites (d-ROM) are a comprehensive marker of biological oxidative modifications in serum. 2021 A prospective study of 517 hemodialysis patients prospectively studied with d-ROM as a marker of oxidative stress, adjusted for the inflammatory marker CRP, found that CRP and d-ROM had predictive value for cardiovascular event occurrence and all-cause mortality, but in HD inflammation appears to be more important in the occurrence of cardiovascular events in patients. This study demonstrates that there appears to be a common causal pathway between inflammation and oxidative stress and that they can contribute to each other (Sasaki et al., 2021).

### Oxidative Stress Under HD Conditions is Associated With Cardiovascular Disease Development

#### Occurrence of Cardiovascular Disease

Inflammation, an unconventional cardiovascular risk factor, primarily accelerates the onset and progression of atherosclerosis. Long-term maintenance hemodialysis was first proposed to accelerate the progression of atherosclerosis by Lindner et al., in 1974 (Lindner et al., 1974). Subsequently, Gerrity et al. suggested that inflammation mediates the formation and progression of atherosclerosis (Gerrity, 1981). Patients with ESRD have a characteristic accelerated atherosclerotic process in which chronic inflammation is critical to the progression of atherosclerosis, and the coronary arteries are the most severely hit in this pathological process (Papagianni et al., 2003; Pencak et al., 2013). CKD is associated with the development of early atherosclerosis and the degree of atherosclerosis increases with the progression of CKD (Valdivielso et al., 2019). The incidence of atherosclerosis and the rate of disease progression were more significant in CKD patients treated with HD (Ma et al., 1992; Fujisawa et al., 2000).

#### Lipid Alterations due to Inflammation and Oxidative Stress

Patients receiving HD have abnormalities in certain lipoproteins (including very low density lipoproteins, low density lipoproteins, and intermediate density lipoproteins), which are associated with changes in the arterial wall (Shoji et al., 1998; Nishizawa et al., 2003). 2017 Echida et al. found that higher serum non-HDL cholesterol levels were significantly associated with cardiovascular mortality (Echida et al., 2012). Oxidative modification of low-density lipoprotein cholesterol (LDL-C) affects the onset and progression of atherosclerosis and may lead to cardiovascular disease (Varan et al., 2010; Hopkins, 2013). Oxidatively modified low-density lipoprotein (Ox-LDL) recruits mononuclear macrophages, and scavenger receptors on the cell surface increase macrophage and vascular smooth muscle cell production through uptake of Ox-LDL, ultimately leading to atherogenic plaque formation (increased foam cell formation) and damage to the endothelium. The presence of more significant lipid oxidation due to increased oxidative stress in HD conditions explains the extremely high risk of cardiovascular disease and poor prognosis in HD patients (Kronenberg et al., 2003).

In addition, as the most abundantly expressed protein product in adipose tissue, lipocalin (ADPN) is involved in the regulation of the neuroendocrine system, can regulate lipid disorders, and is positively correlated with the level of inflammation in the body. In hemodialysis patients, abnormal oxidative stress decreases ADPN secretion. In turn, reduced ADPN levels reduce the clearance of ROS, further exacerbating oxidative stress, causing kidney damage, and increasing the incidence of cardiovascular disease (Yu et al., 2015).

#### Tissue Damage and Endothelial Dysfunction

Atherosclerosis occurs first by macrophages invading the vascular endothelium and transforming into foam cells to form atheromatous material. At the same time, the invading leukocytes release inflammatory mediators that lead to endothelial damage and, in some cases, induce atherosclerotic plaque rupture leading to fatal disease (Liberale et al., 2021). Inflammation and oxidative stress are directly related to the development of cardiovascular disease (CVD) in patients with CKD. Abnormal oxidative stress in ESRD patients can oxidize lipids, proteins and carbohydrates, leading to tissue damage and endothelial dysfunction, exacerbated by the effects of uremic toxins (Dummer et al., 2007; Westerweel et al., 2007).

Serum malondialdehyde (MDA) levels are an important indicator of lipid peroxidation and a strong indicator of cardiovascular disease. Circulating malondialdehyde modified low-density lipoprotein (MDA-LDL) is the main end product of LDL oxidation, negatively correlates with endothelial function, and predicts the onset and progression of atherosclerosis (atherosclerosis and arterial calcification) in the population (Ito et al., 2018). Calcium and phosphorus metabolism is deranged in uremic patients, leading to activated oxidation of fats and proteins and increased MDA production, exacerbating the risk of cardiovascular disease (Hou et al., 2020). Zhang et al. investigated the factors associated with promoting the development of cardiac insufficiency in uremic patients and evaluated CRP and MDA as markers of inflammatory and oxidative stress mechanisms, respectively, and found that CRP and MDA were negatively correlated with left ventricular ejection fraction (LVEF) (Zhang et al., 2021). Second, elevated serum inorganic phosphorus is often observed in CKD patients. The high phosphate environment leads to increased angiogenesis, endothelial cell senescence, apoptosis, and translocation, thereby disrupting endothelial function and promoting the development of vascular calcification in CKD patients (Di Marco et al., 2008; Rapa et al., 2021).

Zinc (Zn), a biological antioxidant, increases oxidative stress *in vivo* in HD patients due to low plasma zinc levels (mainly due to reduced renal function, diminished intestinal absorption of zinc, and exogenous factors such as diet and medications), causing LDL to differentiate toward electronegative LDL [LDL(-)]. the presence of LDL(-) stimulates the production of many of the inflammatory mediators mentioned previously and promotes cardiovascular disease by recruiting leukocytes to cause pathological changes in the vascular endothelium promoting cardiovascular disease.

### Excessive ROS Generation

Patients with chronic kidney disease are constantly exposed to oxidative stress, especially those with ESRD (Witko-Sarsat et al., 1998; Oberg et al., 2004; Tsuchikura et al., 2010). Oxidative stress mechanisms play a more important role in the occurrence of adverse events in HD patients compared to biologic incompatibilities, therefore in uremic patients with HD, increased oxidative stress mechanisms appear to be a more important target for drug and biologic incompatibility therapy in such patients.

Long-term use of AVG and CVC for dialysis in HD patients promotes inflammation and OS due to the metabolic abnormalities associated with uremia and the biologically incompatible system of hemodialysis (Himmelfarb et al., 2002). Biological incompatibility depends on the type of dialysis membrane and the endotoxin dissemination caused by the contaminated dialysate, which activates phagocytes in the blood and leads to the progression of inflammation. At the same time, activated neutrophils and monocytes in the blood produce reactive intermediates that exacerbate oxidative imbalances and further promote inflammation (Tetta et al., 1999; Varan et al., 2010). Hemodialysis activates the complement system, recruiting small molecules such as immunoglobulins (IgG) and complement to attach to the dialysis membrane, thereby promoting the release of ROS (Liakopoulos et al., 2017; Liakopoulos et al., 2019). The above multiple mechanisms explain the induction of oxidative stress and inflammation by biologic incompatibilities, further promoting the development of coronary sclerosis in HD patients (Schettler et al., 1998; Fumeron et al., 2005).

Varan et al. compared the effects of different dialysis membranes on oxidative stress in HD patients and found that all antioxidant enzymes were significantly increased in patients receiving HD treatment. In patients included in the study, even a single dialysis session with a biocompatible membrane (e.g., polysulfone) resulted in a lesser degree of oxidant/antioxidant imbalance *in vivo* than dialysis with a biologically incompatible membrane (e.g, copperane) (Varan et al., 2010).

Angiotensin II, an effector mediating vascular cell hypertrophy, fibrosis, inflammation, and cellular senescence, increases ROS production, and activation of oxidative stress-sensitive transcription factors through activation of nicotinamide adenine dinucleotide phosphate (NADPH) oxidase (Nox) activity and promotes inflammation (Griendling et al., 1994). Nox isoforms (especially Nox1, Nox2, Nox4, and Nox5) are involved in intrarenal oxidative stress and stimulate oxidative stress. Nox2, Nox4, and Nox5) are involved in intrarenal oxidative stress and stimulate oxidative stress, which is common in CKD patients (Madero et al., 2009; Bobulescu, 2010; Liesa and Shirihai, 2013; Sedeek et al., 2013; Gondouin et al., 2015; Ziegler et al., 2015; Gamboa et al., 2016; Wan et al., 2016; Ge et al., 2020; Martínez-Klimova et al., 2020; Rayego-Mateos and Valdivielso, 2020; Aranda-Rivera et al., 2021). Physiological doses of Nox4 are involved in cell proliferation, metabolism, and apoptosis (Sedeek et al., 2013), but excessive concentrations of Nox4 can lead to cellular inflammation, fibrosis, and even cellular damage, which subsequently affects renal excretory function. Many studies have suggested that uric acid can be used as a prognostic indicator for cardiovascular disease and CKD, possibly due to increased xanthine oxidase (XO) activity in patients with hyperuricemia, which leads to oxidative stress and endothelial dysfunction. XO is a pro-oxidant enzyme that promotes ROS production and is involved in the uric acid synthesis (Madero et al., 2009). XO activity is increased in HD patients, which is associated with abnormal oxidative stress in HD patients. Because of the pro-oxidant effect of XO, XO activity can be used as a predictor of cardiovascular events in patients with CKD (Gondouin et al., 2015).

**FIGURE 2:**
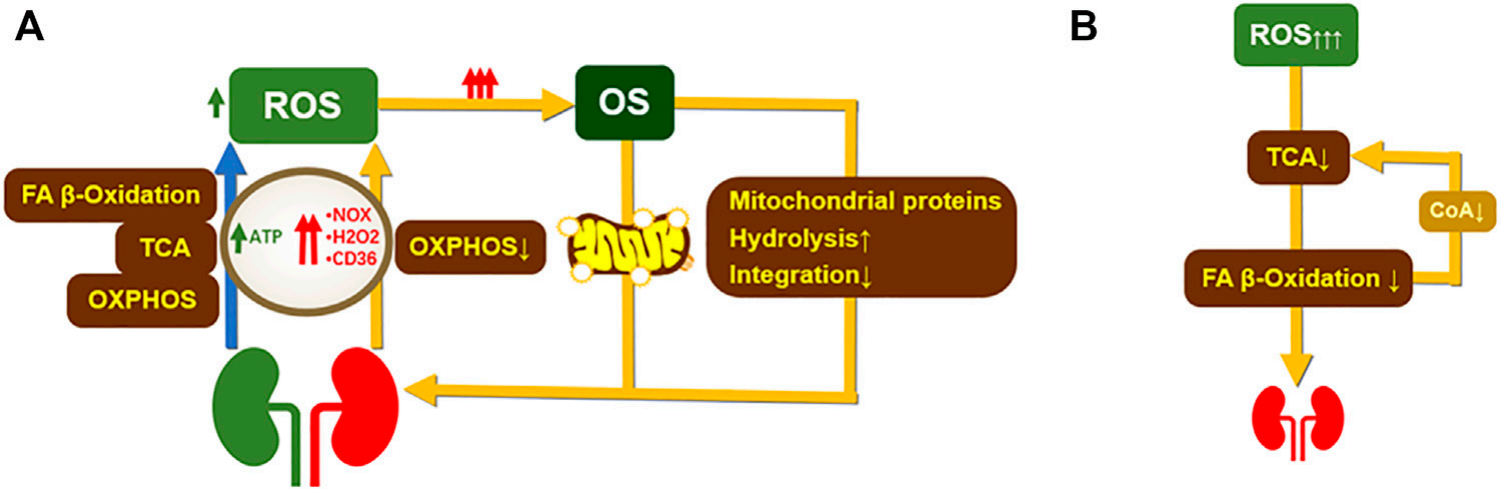
The occurrence of renal oxidative stress affects mitochondrial structure and function. **(A)**. In normal humans, oxidative phosphorylation, tricarboxylic acid cycle, and FA-β-oxidation provide energy to the kidney and induce the production of low doses of ROS. OXPHOS mainly provide ATP to the proximal tubule to maintain the normal physiological function of the kidney. A decrease in OXPHOS capacity during kidney damage leads to an increase in NOX levels. Abnormal NOX levels lead to excessive ROS production exacerbating oxidative stress and inflammation and inducing the development of renal fibrosis. Meanwhile, renal disease impairs mitochondrial function during OXPHOS due to reduced renal degradation of H_2_O_2_ a FA-βnd disrupts FA-β oxidation. overexpression of CD36 leads to reduced lipid metabolism resulting in involvement and causes impaired FA-β oxidation, leading to excessive ROS production (Ge et al., 2020; Martínez-Klimova et al., 2020). Excess ROS leads to growth mitochondrial damage, 1) decreased mitochondrial protein synthesis, and increased catabolism (Liesa and Shirihai, 2013). 2) Increased mitochondrial autophagy, which occurs mainly through degradation of proteasomes by the PINK1-Parkin pathway and phagocytic receptor interactions. **(B)**. Excess ROS impair FA-β-oxidation by impairing TCA cycle function. reduced FA-β-oxidation leads to lipid accumulation and aggravates renal function. In turn, impaired FA *β*-oxidation leads to a decrease in acetyl coenzyme A, further diminishing TCA capacity (Bobulescu, 2010). ROS, reactive oxygen species; OS, oxidative stress; OXPHOS, oxidative phosphorylation; TCA, tricarboxylic acid cycle; NOX, nicotinamide adenine dinucleotide phosphate (NADPH) oxidase; H_2_O_2_, hydrogen peroxide; CD36, long-chain FA fractionation cluster36.

Under physiological conditions, mitochondria maintain normal cellular signaling pathways through fatty acid (FA) *β*-oxidation, tricarboxylic acid cycle (TCA cycle), oxidative phosphorylation (OXPHOS) to produce adenosine triphosphate (ATP), and small doses of ROS, and ATP is also involved in active transport in the renal tubules to maintain normal physiological functions. Abnormalities in many genes related to protein production and mitochondrial activity can be observed in patients on renal replacement therapy (Rayego-Mateos and Valdivielso, 2020; Gamboa et al., 2016). In addition to this, impaired FA *β*-oxidation and OXPHOS reduce ATP synthesis and increase NOX, hydrogen peroxide (H_2_O_2_), and CD36 levels in the body, inducing excessive ROS production and stimulating the onset of oxidative stress. The pathological increase of oxidative stress induces the occurrence of mitochondrial autophagy and weakens mitochondrial protein synthesis, further deteriorating mitochondrial function and destroying renal compensatory function (Ziegler et al., 2015). In turn, a pathological increase in ROS further inhibits ATP synthesis, while a decrease in ATP synthesis positively increases ROS production, leading to increased oxidative stress and destruction of cellular components resulting in cell necrosis (Figure 2) (Aranda-Rivera et al., 2021). Therefore, balancing the redox process can improve mitochondrial function and optimize mitochondrial dynamics by applying antioxidants that target mitochondria.

### Anti-Oxidative Stress

Under physiological conditions, free oxygen radicals act as a non-specific defense mechanism against stimuli such as infections. However, when overproduced, they are harmful to the body, affecting lipids, proteins, DNA, and cell membranes, including membrane carbohydrates, leading to membrane damage and cell disintegration. To prevent or mitigate the adverse effects of ROS, antioxidant mechanisms play an important role (Aiello et al., 1999). Uremic patients have a significant disruption of the extracellular redox system, with a tendency toward increased oxidative status and antioxidant depletion, which is exacerbated by HD. ESRD patients treated with HD, therefore, require a more active antioxidant system, yet the available clinical evidence suggests that such patients have an antioxidant dysfunction (Varan et al., 2010). Therefore, the appropriate use of antioxidants in ESRD patients may prevent renal injury and disease progression and reduce the incidence of adverse events by modulating oxidative stress.

Multiple antioxidant stress substances are affected in CKD, especially in HD patients. SOD isomers are important internal enzymes against oxidative stress, and SOD-1, an important isomer of SOD in the kidney, is associated with impaired renal function and reduced renal excretion capacity, so increased SOD-1 synthesis is observed in HD patients (Pawlak et al., 2007; Pawlak et al., 2013). In addition, SOD-1 can be used as a marker of atherosclerosis, vascular abnormalities, and inflammation. However, due to its influence by various factors such as immune status and enzyme activity, the current studies have not reached consistent conclusions about SOD-1. Renal glutathione peroxidase 1 (GPx1) can prevent some damage to the kidney caused by oxidative stress and enhance the kidney’s ability to cope with oxidative stress, so proper induction of such enzyme activity and promotion of its gene expression may be clinically beneficial for ESRD patients (Chu et al., 2020). In addition, patients with advanced kidney disease suffer from micronutrient dysregulation due to dietary restrictions, poor appetite, and dialysis depletion, resulting in antioxidant deficiency and insufficient activity. A meta-analysis evaluating the effect of multiple oxidants on all-cause mortality and cardiovascular event rates showed that antioxidants reduced the incidence of cardiovascular events in HD patients without a significant effect on non-dialysis patients (Jun et al., 2012; Baldi et al., 2013; Sepe et al., 2019).

Several studies have confirmed that active components involved in oxidative stress can improve the poor prognosis of HD patients (Table 3). In addition, quercetin, amino acids, tea-lipoic acid, and lipocalin are involved in the regulation of oxidative stress.

**TABLE 3:** Protective effects of common antioxidant substances on cardiac function in dialysis patients.

Antioxidants	Renal protection mechanism
Taurine	Scavenges ROS, reduces inflammatory response, plays a role in phagocytosis and reduces inflammation, and protects against hemodialysis, ischemia, and various renal diseases
I-Carnitine	Reducing the production of acetyl coenzyme a and thus the production of free radicals reduces the production of pro-inflammatory factors in dialysis patients and is beneficial to the kidney
Vitamin C and Vitamin E	In dialysis patients, oxidative stress is associated with reduced vitamin C (Mirna et al., 2020). Infusion of vitamin C and use of vitamin E coated dialyzers in HD patients attenuates oxidative stress by reducing IDO1 activity and NO formation, weakening inflammation and cellular senescence (Januzzi et al., 2015). Vitamin E supplementation may reduce the risk of coronary artery disease by making LDL less susceptible to oxidation (Gaggin and Januzzi, 2013)
Niacinamide	Reduces the production of many cytokines associated with the pathogenesis of cardiac insufficiencies, such as IL-1β, IL-6, IL-8, and tumor necrosis factor

### Medications for Inflammation Control

Drugs commonly used to treat cardiovascular disease may have a higher cardiovascular benefit for ESRD patients due to their potential anti-inflammatory effects (Fiorillo et al., 1998; Helmke and von Vietinghoff, 2016). We have listed the commonly used cardiovascular drugs with anti-inflammatory effects, but there are a variety of drugs with anti-inflammatory effects including colchicine, methotrexate, and interleukin receptor antagonists, and their association with CKD has been little studied and not discussed.

### Statins

There is no clear evidence to support the benefit of anti-inflammatory therapy in the dialysis population with cardiac insufficiency, and statins may provide a benefit in the treatment and prevention of cardiovascular disease due to their LDL-lowering and anti-inflammatory effects, but there is no clear evidence for patients on dialysis. The study by Baigent et al. concluded that the use of statins and ezetimibe in combination with lipid-lowering therapy did not result in a better prognosis for dialysis patients compared to non-users. It was also suggested that there was no significant correlation between LDL and cardiovascular prognosis in dialysis patients (Baigent and Landry, 2003). Antibodies to pro-inflammatory cytokines may reduce the risk of adverse cardiovascular events, particularly in patients with CRP <2 mg/L, and anti-inflammatory therapy may be indicated at the start of dialysis, but more clinical findings are needed to support this (Dhorepatil et al., 2019). A 2021 study of a population with coronary heart disease using statins for more than 2 years showed that statins caused a gradual shift to higher density calcification of coronary atherosclerotic plaques while attenuating the progression of coronary atherosclerotic plaque size, but the explanation for the acceleration of coronary atherosclerotic plaque calcification by statins remains controversial (van Rosendael et al., 2021).

### PCSK9 Inhibitor

Proprotein convertase subtilisin-kexin type 9 (PCSK9) is the main carrier of LDL-C and causes elevated LDL-C levels, which are associated with inflammation and immunity. Therefore, PCSK9 inhibitors not only reduce LDL-C, but also modulate inflammation and autoimmunity (Zhang et al., 2007). Therefore, PCSK9 inhibitors not only lower LDL-C, but also regulate inflammation and autoimmunity (Frostegård, 2021).

In a study of 9,738 people at high cardiovascular risk receiving statins and PCSK9 inhibitors, Pradhan et al. found that PCSK9 inhibitors significantly lowered LDL-C while reducing hs-CRP by 6.6%. hs-CRP >3 mg/L suggests a high risk of cardiovascular disease and kidney disease (Adejumo et al., 2016). Notably, despite the maximal reduction of LDL in this study, the presence of residual inflammatory risk (hsCRP ≥ 2 mg/L) still put the risk of future cardiovascular disease at a higher risk, whereas the use of PCSK9 inhibitors significantly reduced the risk of cardiovascular events in the high-risk population. This phenomenon cannot be explained by lipid-lowering effects alone, which may be due to the effect of PCSK9 inhibitors on inflammatory regulation in high-risk populations *in vivo* (Pradhan et al., 2018). The FOURIER trial then investigated the efficacy of PCSK9 inhibition in patients with stable coronary artery disease in different hs-CRP strata and found that the positive cardiovascular effects of PCSK9 inhibition were certain regardless of baseline hs-CRP, with patients with higher baseline hs-CRP showing the greatest benefit with PCSK9 inhibition (Bohula et al., 2018).

PSCK9 may serve as a novel cardiovascular risk marker in ESRD patients.2019 Strålberg et al. found that PCSK-9 levels were independently associated with all-cause mortality in 265 ESRD patients receiving long-term HD at a 3-year follow-up (Strålberg et al., 2019). 2021 Vlad et al. found no significant difference in PCSK9 levels in patients with different CKD stages, but PCSK9 > 220 ng/ml was a predictor of cardiovascular events, and PCSK9 > 220 ng/ml and hsCRP >3 mg/L together suggested an increased risk of kidney disease and cardiovascular disease (Vlad et al., 2021). However, PCSK9 inhibitors are less commonly used in patients with ESRD, and their efficacy in such patients is unclear.

### Aspirin

The role of aspirin, a drug that provides great benefit for the treatment and prevention of cardiovascular disease, is not promising in patients with CKD. A cross-sectional study of 116 long-term aspirin users in the general population found that CKD patients were more likely to have impaired antiplatelet effects, and that such impaired effects were associated with increased mortality (Mayer et al., 2014; Polzin et al., 2016). A 2016 meta-analysis by Major et al. noted that aspirin did not provide benefit for cardiovascular events as well as prevention in CKD patients, nor did it improve survival in CKD patients, and may also carry a higher risk of major bleeding (Major et al., 2016). A controlled study of 17,762 subjects using aspirin or placebo in 2020 found a significantly increased risk of CVD in 4,768 participants with CKD and that the use of aspirin did not reduce the incidence of CVD in patients with CKD (Wolfe et al., 2021). 2021 et al. studied 91,744 ESRD patients who were not on dialysis and showed that aspirin caused disease progression and increased mortality in these patients (Tsai et al., 2021). Overall, aspirin does more harm than good in patients with ESRD. The ongoing ATTACK trial is the first trial to evaluate aspirin for the prevention of primary CVD in adults with CKD, and this large study may bring more reliable evidence for the use of aspirin in patients with CKD.

## Conclusion

Cardiovascular complications in patients with HD are a fatal factor in patients with ESRD. Inflammation is involved in and mediates the development of cardiovascular complications. While hemodialysis benefits the kidney, it exacerbates an already inflammatory state, altering the body’s already tolerated microinflammation and volume overload, leading to a “cardiovascular spike”. At the same time, the oxidative stress that accompanies inflammation during HD treatment damages renal structures, leading to further deterioration of renal function and increasing the risk of cardiovascular disease. Disturbances in electrolyte and acid-base balance secondary to the deterioration of renal function feedback into the oxidative stress mechanism, leading to a vicious circle between oxidative stress and the kidney. This review details the mechanisms of inflammation in ESRD patients treated with HD, the impact of different processes in the inflammatory cascade on cardiovascular complications, the mechanisms of oxidative stress, and the correlation between oxidative stress and cardiovascular disease. Although the mechanisms of inflammation are vast and complex, as a key factor in the development and prognosis of cardiovascular complications in ESRD patients, interventions that address key targets in the inflammatory cascade (e.g., immune mechanisms, complement activation, etc.) may improve compliance with HD therapy in ESRD patients and provide additional clinical benefits.

## References

[B1] Adda-Rezig H. Carron C. Pais de Barros J. P. Choubley H. Charron É. Rérole A. L. (2021). New Insights on End-Stage Renal Disease and Healthy Individual Gut Bacterial Translocation: Different Carbon Composition of Lipopolysaccharides and Different Impact on Monocyte Inflammatory Response. Front. Immunol. 12, 658404. 10.3389/fimmu.2021.658404 34163471 PMC8215383

[B2] Adejumo O. A. Okaka E. I. Okwuonu C. G. Iyawe I. O. Odujoko O. O. (2016). Serum C-Reactive Protein Levels in Pre-dialysis Chronic Kidney Disease Patientsin Southern Nigeria. Ghana Med. J. 50 (1), 31–38. 10.4314/gmj.v50i1.5 27605722 PMC4994480

[B3] Ahbap E. Sakaci T. Kara E. Sahutoglu T. Koc Y. Basturk T. (2016). Neutrophil-to-lymphocyte Ratio and Platelet-Tolymphocyte Ratio in Evaluation of Inflammation in End-Stage Renal Disease. Clin. Nephrol. 85 (4), 199–208. 10.5414/CN108584 26521887

[B4] Aiello S. Noris M. RemuzziNitric G. (1999). Nitric oxide/L-Arginine in Uremia. Miner Electrolyte Metab. 25 (4-6), 384–390. 10.1159/000057479 10681671

[B5] Alam M. L. Katz R. Bellovich K. A. Bhat Z. Y. Brosius F. C. de Boer I. H. (2019). Soluble ST2 and Galectin-3 and Progression of CKD. Kidney Int. Rep. 4 (1), 103–111. 10.1016/j.ekir.2018.09.013 30596173 PMC6308819

[B6] Allison S. J. (2016). Chronic Kidney Disease: suPAR in CKD. Nat. Rev. Nephrol. 12 (1), 3. 10.1038/nrneph.2015.195 26592192

[B7] Ao G. Wang Y. Qi X. Wang F. Wen H. (2021). Association of Neutrophil-To-Lymphocyte Ratio and Risk of Cardiovascular or All-Cause Mortality in Chronic Kidney Disease: a Meta-Analysis. Clin. Exp. Nephrol. 25 (2), 157–165. 10.1007/s10157-020-01975-9 33025234

[B8] Aranda-Rivera A. K. Cruz-Gregorio A. Aparicio-Trejo O. E. Pedraza-Chaverri J. (2021). Mitochondrial Redox Signaling and Oxidative Stress in Kidney Diseases. Biomolecules 11 (8). 10.3390/biom11081144 PMC8391472 34439810

[B9] Arcari L. Ciavarella G. M. Altieri S. Limite L. R. Russo D. Luciani M. (2020). Longitudinal Changes of Left and Right Cardiac Structure and Function in Patients with End-Stage Renal Disease on Replacement Therapy. Eur. J. Intern. Med. 78, 95–100. 10.1016/j.ejim.2020.04.051 32402562

[B10] Arcari L. Engel J. Freiwald T. Zhou H. Zainal H. Gawor M. (2021). Cardiac Biomarkers in Chronic Kidney Disease Are Independently Associated with Myocardial Edema and Diffuse Fibrosis by Cardiovascular Magnetic Resonance. J. Cardiovasc. Magn. Reson. 23 (1), 71. 10.1186/s12968-021-00762-z 34092229 PMC8183054

[B11] Assimon M. M. Wang L. Flythe J. E. (2018). Failed Target Weight Achievement Associates with Short-Term Hospital Encounters Among Individuals Receiving Maintenance Hemodialysis. J. Am. Soc. Nephrol. 29 (8), 2178–2188. 10.1681/ASN.2018010004 29793962 PMC6065090

[B12] Babaei M. Dashti N. Lamei N. Abdi K. Nazari F. Abbasian S. (2014). Evaluation of Plasma Concentrations of Homocysteine, IL-6, TNF-Alpha, Hs-CRP, and Total Antioxidant Capacity in Patients with End-Stage Renal Failure. Acta Med. Iran 52 (12), 893–898. 25530051

[B13] Baigent C. Landry M. (2003). Study of Heart and Renal Protection (SHARP). Kidney Int. Suppl. 1 (84), S207–S210. 10.1046/j.1523-1755.63.s84.4.x 12694346

[B14] Baldi S. Innocenti M. Frascerra S. Nannipieri M. Lippi A. Rindi P. (2013). Effects of Hemodialysis and Vitamin E Supplementation on Low-Density Lipoprotein Oxidizability in End-Stage Renal Failure. J. Nephrol. 26 (3), 549–555. 10.5301/jn.5000190 22941872

[B15] Banerjee T. Kim S. J. Astor B. Shafi T. Coresh J. Powe N. R. (2014). Vascular Access Type, Inflammatory Markers, and Mortality in Incident Hemodialysis Patients: the Choices for Healthy Outcomes in Caring for End-Stage Renal Disease (CHOICE) Study. Am. J. Kidney Dis. 64 (6), 954–961. 10.1053/j.ajkd.2014.07.010 25266479 PMC4265216

[B16] Bangalore S. Maron D. J. O'Brien S. M. Fleg J. L. Kretov E. I. Briguori C. (2020). Management of Coronary Disease in Patients with Advanced Kidney Disease. N. Engl. J. Med. 382 (17), 1608–1618. 10.1056/NEJMoa1915925 32227756 PMC7274537

[B17] Barreto D. V. Barreto F. C. Liabeuf S. Temmar M. Lemke H. D. Tribouilloy C. (2010). Plasma Interleukin-6 Is Independently Associated with Mortality in Both Hemodialysis and Pre-dialysis Patients with Chronic Kidney Disease. Kidney Int. 77 (6), 550–556. 10.1038/ki.2009.503 20016471

[B18] Bobulescu I. A. (2010). Renal Lipid Metabolism and Lipotoxicity. Curr. Opin. Nephrol. Hypertens. 19 (4), 393–402. 10.1097/MNH.0b013e32833aa4ac 20489613 PMC3080272

[B19] Bohula E. A. Giugliano R. P. Leiter L. A. Verma S. Park J. G. Sever P. S. (2018). Inflammatory and Cholesterol Risk in the FOURIER Trial. Circulation 138 (2), 131–140. 10.1161/CIRCULATIONAHA.118.034032 29530884

[B20] Boltuc K. Bociek A. Dziugiel R. Bociek M. Zapolski T. Dabrowski W. (2020). Neutrophil-Lymphocyte Ratio (NLR) Reflects Myocardial Inhomogeneities in Hemodialyzed Patients. Mediators Inflamm. 2020, 6027405. 32963494 10.1155/2020/6027405 PMC7486637

[B21] Borné Y. Persson M. Melander O. Smith J. G. Engström G. (2014). Increased Plasma Level of Soluble Urokinase Plasminogen Activator Receptor Is Associated with Incidence of Heart Failure but Not Atrial Fibrillation. Eur. J. Heart Fail. 16 (4), 377–383. 10.1002/ejhf.49 24464777

[B22] Braunwald E. Kloner R. A. (1982). The Stunned Myocardium: Prolonged, Postischemic Ventricular Dysfunction. Circulation 66 (6), 1146–1149. 10.1161/01.cir.66.6.1146 6754130

[B23] Brown-Tortorici A. R. Naderi N. Tang Y. Park C. You A. S. Norris K. C. (2020). Serum Albumin Is Incrementally Associated with Increased Mortality across Varying Levels of Kidney Function. Nutrition 79-80, 110818. 10.1016/j.nut.2020.110818 32634605

[B24] Buraczynska M. Ksiazek P. Zukowski P. Benedyk-Lorens E. Orlowska-Kowalik G. (2009). Complement Factor H Gene Polymorphism and Risk of Cardiovascular Disease in End-Stage Renal Disease Patients. Clin. Immunol. 132 (2), 285–290. 10.1016/j.clim.2009.04.005 19428307

[B25] Cao H. Ye H. Sun Z. Shen X. Song Z. Wu X. (2014). Circulatory Mitochondrial DNA Is a Pro-inflammatory Agent in Maintenance Hemodialysis Patients. PLoS One 9 (12), e113179. 10.1371/journal.pone.0113179 25485699 PMC4259325

[B26] Chan C. T. Blankestijn P. J. Dember L. M. Gallieni M. Harris D. C. H. Lok C. E. (2019). Dialysis Initiation, Modality Choice, Access, and Prescription: Conclusions from a Kidney Disease: Improving Global Outcomes (KDIGO) Controversies Conference. Kidney Int. 96 (1), 37–47. 10.1016/j.kint.2019.01.017 30987837

[B27] Chan K. Moe S. M. Saran R. Libby P. (2021). The Cardiovascular-Dialysis Nexus: the Transition to Dialysis Is a Treacherous Time for the Heart. Eur. Heart J. 42 (13), 1244–1253. 10.1093/eurheartj/ehaa1049 33458768 PMC8014523

[B28] Cheung A. K. Chang T. I. Cushman W. C. Furth S. L. Hou F. F. Ix J. H. (2021). Executive Summary of the KDIGO 2021 Clinical Practice Guideline for the Management of Blood Pressure in Chronic Kidney Disease. Kidney Int. 99 (3), 559–569. 10.1016/j.kint.2020.10.026 33637203

[B29] Chu Y. Lan R. S. Huang R. Feng H. Kumar R. Dayal S. (2020). Glutathione Peroxidase-1 Overexpression Reduces Oxidative Stress, and Improves Pathology and Proteome Remodeling in the Kidneys of Old Mice. Aging Cell 19 (6), e13154. 10.1111/acel.13154 32400101 PMC7294784

[B30] Colì L. Tumietto F. De Pascalis A. La Manna G. Zanchelli F. Isola E. (1999). Effects of Dialysis Membrane Nature on Intradialytic Phagocytizing Activity. Int. J. Artif. Organs 22 (2), 74–80. 10.1177/039139889902200204 10212041

[B31] Crépin T. Legendre M. Courivaud C. Vauchy C. Laheurte C. Rebibou J. M. (2020). [Premature Immune Senescence and Chronic Kidney Disease: Update and Perspectives]. Nephrol. Ther. 16 (1), 9–18. 10.1016/j.nephro.2019.04.005 31848067

[B32] Crotty S. (2019). T Follicular Helper Cell Biology: A Decade of Discovery and Diseases. Immunity 50 (5), 1132–1148. 10.1016/j.immuni.2019.04.011 31117010 PMC6532429

[B33] Dalal J. J. Digrajkar A. Das B. Bansal M. Toomu A. Maisel A. S. (2018). ST2 Elevation in Heart Failure, Predictive of a High Early Mortality. Indian Heart J. 70 (6), 822–827. 10.1016/j.ihj.2018.08.019 30580851 PMC6306350

[B34] deFilippi C. Seliger S. L. Kelley W. Duh S. H. Hise M. Christenson R. H. (2012). Interpreting Cardiac Troponin Results from High-Sensitivity Assays in Chronic Kidney Disease without Acute Coronary Syndrome. Clin. Chem. 58 (9), 1342–1351. 10.1373/clinchem.2012.185322 22791885

[B35] dell'Oglio M. P. Simone S. Ciccone M. Corciulo R. Gesualdo M. Zito A. (2017). Neutrophil-dependent Pentraxin-3 and Reactive Oxygen Species Production Modulate Endothelial Dysfunction in Haemodialysis Patients. Nephrol. Dial. Transpl. 32 (9), 1540–1549. 10.1093/ndt/gfw363 27915246

[B36] Dhorepatil A. Ball S. Ghosh R. K. Kondapaneni M. Lavie C. J. (2019). Canakinumab: Promises and Future in Cardiometabolic Diseases and Malignancy. Am. J. Med. 132 (3), 312–324. 10.1016/j.amjmed.2018.10.013 30832770

[B37] Di Marco G. S. Hausberg M. Hillebrand U. Rustemeyer P. Wittkowski W. Lang D. (2008). Increased Inorganic Phosphate Induces Human Endothelial Cell Apoptosis *In Vitro* . Am. J. Physiol. Ren. Physiol 294 (6), F1381–F1387. 10.1152/ajprenal.00003.2008 18385273

[B38] Dummer C. D. Thomé F. S. Veronese F. V. (2007). [Chronic Renal Disease, Inflammation and Atherosclerosis: New Concepts about an Old Problem]. Rev. Assoc. Med. Bras (1992) 53 (5), 446–450. 10.1590/s0104-42302007000500022 17952355

[B39] Düsing P. Zietzer A. Goody P. R. Hosen M. R. Kurts C. Nickenig G. (2021). Vascular Pathologies in Chronic Kidney Disease: Pathophysiological Mechanisms and Novel Therapeutic Approaches. J. Mol. Med. (Berl) 99 (3), 335–348. 10.1007/s00109-021-02037-7 33481059 PMC7900031

[B40] Echida Y. Ogawa T. Otsuka K. Ando Y. Nitta K. (2012). Serum Non-high-density Lipoprotein Cholesterol (Non-HDL-C) Levels and Cardiovascular Mortality in Chronic Hemodialysis Patients. Clin. Exp. Nephrol. 16 (5), 767–772. 10.1007/s10157-012-0615-5 22374049

[B41] Esposito P. Rampino T. Gregorini M. Gabanti E. Bianzina S. Dal Canton A. (2012). Mechanisms Underlying sCD40 Production in Hemodialysis Patients. Cell Immunol 278 (1-2), 10–15. 10.1016/j.cellimm.2012.06.007 23121970

[B42] Ethier J. Mendelssohn D. C. Elder S. J. Hasegawa T. Akizawa T. Akiba T. (2008). Vascular Access Use and Outcomes: an International Perspective from the Dialysis Outcomes and Practice Patterns Study. Nephrol. Dial. Transpl. 23 (10), 3219–3226. 10.1093/ndt/gfn261 PMC2542410 18511606

[B43] Eugen-Olsen J. Andersen O. Linneberg A. Ladelund S. Hansen T. W. Langkilde A. (2010). Circulating Soluble Urokinase Plasminogen Activator Receptor Predicts Cancer, Cardiovascular Disease, Diabetes and Mortality in the General Population. J. Intern. Med. 268 (3), 296–308. 10.1111/j.1365-2796.2010.02252.x 20561148

[B44] Fernandez D. M. Rahman A. H. Fernandez N. F. Chudnovskiy A. Amir E. D. Amadori L. (2019). Single-cell Immune Landscape of Human Atherosclerotic Plaques. Nat. Med. 25 (10), 1576–1588. 10.1038/s41591-019-0590-4 31591603 PMC7318784

[B45] Ferraccioli G. Gremese E. (2017). B Cell Activating Factor (BAFF) and BAFF Receptors: Fakes and Facts. Clin. Exp. Immunol. 190 (3), 291–292. 10.1111/cei.13039 28834574 PMC5680055

[B46] Fiorillo C. Oliviero C. Rizzuti G. Nediani C. Pacini A. Nassi P. (1998). Oxidative Stress and Antioxidant Defenses in Renal Patients Receiving Regular Haemodialysis. Clin. Chem. Lab. Med. 36 (3), 149–153. 10.1515/CCLM.1998.028 9589802

[B47] Flythe J. E. Chang T. I. Gallagher M. P. Lindley E. Madero M. Sarafidis P. A. (2020). Blood Pressure and Volume Management in Dialysis: Conclusions from a Kidney Disease: Improving Global Outcomes (KDIGO) Controversies Conference. Kidney Int. 97 (5), 861–876. 10.1016/j.kint.2020.01.046 32278617 PMC7215236

[B48] Flythe J. E. Xue H. Lynch K. E. Curhan G. C. Brunelli S. M. (2015). Association of Mortality Risk with Various Definitions of Intradialytic Hypotension. J. Am. Soc. Nephrol. 26 (3), 724–734. 10.1681/ASN.2014020222 25270068 PMC4341481

[B49] Formanowicz D. Wanic-Kossowska M. Pawliczak E. Radom M. Formanowicz P. (2015). Usefulness of Serum Interleukin-18 in Predicting Cardiovascular Mortality in Patients with Chronic Kidney Disease-Ssystems and Clinical Approach. Sci. Rep. 5, 18332. 10.1038/srep18332 26669254 PMC4680867

[B50] Friedman A. N. Fadem S. Z. (2010). Reassessment of Albumin as a Nutritional Marker in Kidney Disease. J. Am. Soc. Nephrol. 21 (2), 223–230. 10.1681/ASN.2009020213 20075063

[B51] Frostegård J. (2021). The Role of PCSK9 in Inflammation, Immunity, and Autoimmune Diseases. Expert Rev. Clin. Immunol. 1, 1–8. 10.1080/1744666X.2022.2017281 34928185

[B52] Fujisawa M. Haramaki R. Miyazaki H. Imaizumi T. Okuda S. (2000). Role of Lipoprotein (A) and TGF-Beta 1 in Atherosclerosis of Hemodialysis Patients. J. Am. Soc. Nephrol. 11 (10), 1889–1895. 10.1681/ASN.V11101889 11004220

[B53] Fukushi T. Yamamoto T. Yoshida M. Fujikura E. Miyazaki M. Nakayama M. (2020). Enhanced Neutrophil Apoptosis Accompanying Myeloperoxidase Release during Hemodialysis. Sci. Rep. 10 (1), 21747. 10.1038/s41598-020-78742-z 33303892 PMC7728788

[B54] Fumeron C. Nguyen-Khoa T. Saltiel C. Kebede M. Buisson C. Drüeke T. B. (2005). Effects of Oral Vitamin C Supplementation on Oxidative Stress and Inflammation Status in Haemodialysis Patients. Nephrol. Dial. Transpl. 20 (9), 1874–1879. 10.1093/ndt/gfh928 15972322

[B55] Gaggin H. K. Januzzi J. L. Jr. (2013). Biomarkers and Diagnostics in Heart Failure. Biochim. Biophys. Acta 1832 (12), 2442–2450. 10.1016/j.bbadis.2012.12.014 23313577

[B56] Gamboa J. L. Billings F. T. Bojanowski M. T. Gilliam L. A. Yu C. Roshanravan B. (2016). Mitochondrial Dysfunction and Oxidative Stress in Patients with Chronic Kidney Disease. Physiol. Rep. 4 (9), e12780. 10.14814/phy2.12780 27162261 PMC4873632

[B57] Ge M. Fontanesi F. Merscher S. Fornoni A. (2020). The Vicious Cycle of Renal Lipotoxicity and Mitochondrial Dysfunction. Front. Physiol. 11, 732. 10.3389/fphys.2020.00732 32733268 PMC7358947

[B58] George R. P. Mehta A. K. Perez S. D. Winterberg P. Cheeseman J. Johnson B. (2017). Premature T Cell Senescence in Pediatric CKD. J. Am. Soc. Nephrol. 28 (1), 359–367. 10.1681/ASN.2016010053 27413076 PMC5198283

[B59] Gerrity R. G. (1981). The Role of the Monocyte in Atherogenesis: I. Transition of Blood-Borne Monocytes into Foam Cells in Fatty Lesions. Am. J. Pathol. 103 (2), 181–190. 7234961 PMC1903817

[B60] Girndt M. Heisel O. Köhler H. (1999). Influence of Dialysis with Polyamide vs Haemophan Haemodialysers on Monokines and Complement Activation during a 4-month Long-Term Study. Nephrol. Dial. Transpl. 14 (3), 676–682. 10.1093/ndt/14.3.676 10193818

[B61] Gondouin B. Jourde-Chiche N. Sallee M. Dou L. Cerini C. Loundou A. (2015). Plasma Xanthine Oxidase Activity Is Predictive of Cardiovascular Disease in Patients with Chronic Kidney Disease, Independently of Uric Acid Levels. Nephron 131 (3), 167–174. 10.1159/000441091 26426087

[B62] Griendling K. K. Minieri C. A. Ollerenshaw J. D. Alexander R. W. (1994). Angiotensin II Stimulates NADH and NADPH Oxidase Activity in Cultured Vascular Smooth Muscle Cells. Circ. Res. 74 (6), 1141–1148. 10.1161/01.res.74.6.1141 8187280

[B63] Guiteras R. Flaquer M. Cruzado J. M. (2016). Macrophage in Chronic Kidney Disease. Clin. Kidney J. 9 (6), 765–771. 10.1093/ckj/sfw096 27994852 PMC5162417

[B64] Gupta J. Mitra N. Kanetsky P. A. Devaney J. Wing M. R. Reilly M. (2012). Association between Albuminuria, Kidney Function, and Inflammatory Biomarker Profile in CKD in CRIC. Clin. J. Am. Soc. Nephrol. 7 (12), 1938–1946. 10.2215/CJN.03500412 23024164 PMC3513744

[B65] Hartzell S. Bin S. Cantarelli C. Haverly M. Manrique J. Angeletti A. (2020). Kidney Failure Associates with T Cell Exhaustion and Imbalanced Follicular Helper T Cells. Front. Immunol. 11, 583702. 10.3389/fimmu.2020.583702 33117396 PMC7552886

[B66] Hayek S. S. Sever S. Ko Y. A. Trachtman H. Awad M. Wadhwani S. (2015). Soluble Urokinase Receptor and Chronic Kidney Disease. N. Engl. J. Med. 373 (20), 1916–1925. 10.1056/NEJMoa1506362 26539835 PMC4701036

[B67] Helmke A. von Vietinghoff S. (2016). Extracellular Vesicles as Mediators of Vascular Inflammation in Kidney Disease. World J. Nephrol. 5 (2), 125–138. 10.5527/wjn.v5.i2.125 26981436 PMC4777783

[B68] Hempel J. C. Poppelaars F. Gaya da Costa M. Franssen C. F. de Vlaam T. P. Daha M. R. (2017). Distinct *In Vitro* Complement Activation by Various Intravenous Iron Preparations. Am. J. Nephrol. 45 (1), 49–59. 10.1159/000451060 27889746

[B69] Herselman M. Esau N. Kruger J. M. Labadarios D. Moosa M. R. (2010). Relationship between Serum Protein and Mortality in Adults on Long-Term Hemodialysis: Exhaustive Review and Meta-Analysis. Nutrition 26 (1), 10–32. 10.1016/j.nut.2009.07.009 20005464

[B70] Himmelfarb J. Stenvinkel P. Ikizler T. A. Hakim R. M. (2002). The Elephant in Uremia: Oxidant Stress as a Unifying Concept of Cardiovascular Disease in Uremia. Kidney Int. 62 (5), 1524–1538. 10.1046/j.1523-1755.2002.00600.x 12371953

[B71] Hodges G. W. Bang C. N. Wachtell K. Eugen-Olsen J. Jeppesen J. L. (2015). suPAR: A New Biomarker for Cardiovascular Disease? Can. J. Cardiol. 31 (10), 1293–1302. 10.1016/j.cjca.2015.03.023 26118447

[B72] Hoenich N. A. Levett D. Fawcett S. Woffindin C. Kerr D. N. (1986). Biocompatibility of Haemodialysis Membranes. J. Biomed. Eng. 8 (1), 3–8. 10.1016/0141-5425(86)90024-5 3512914

[B73] Honkanen E. Grönhagen-Riska C. Teppo A. M. Maury C. P. Meri S. (1991). Acute-phase Proteins during Hemodialysis: Correlations with Serum Interleukin-1 Beta Levels and Different Dialysis Membranes. Nephron 57 (3), 283–287. 10.1159/000186276 2017267

[B74] Hopkins P. N. (2013). Molecular Biology of Atherosclerosis. Physiol. Rev. 93 (3), 1317–1542. 10.1152/physrev.00004.2012 23899566

[B75] Hornum M. Bay J. T. Clausen P. Melchior Hansen J. Mathiesen E. R. Feldt-Rasmussen B. (2014). High Levels of Mannose-Binding Lectin Are Associated with Lower Pulse Wave Velocity in Uraemic Patients. BMC Nephrol. 15, 162. 10.1186/1471-2369-15-162 25281004 PMC4197330

[B76] Hou J. S. Wang C. H. Lai Y. H. Kuo C. H. Lin Y. L. Hsu B. G. (2020). Serum Malondialdehyde-Modified Low-Density Lipoprotein Is a Risk Factor for Central Arterial Stiffness in Maintenance Hemodialysis Patients. Nutrients 12 (7), 2160. 10.3390/nu12072160 PMC7401258 32708072

[B77] Hsiung J. T. Kleine C. E. Naderi N. Park C. Soohoo M. Moradi H. (2019). Association of Pre-end-stage Renal Disease Serum Albumin with Post-End-Stage Renal Disease Outcomes Among Patients Transitioning to Dialysis. J. Ren. Nutr. 29 (4), 310–321. 10.1053/j.jrn.2018.09.004 30642656 PMC6697162

[B78] Huai Q. Mazar A. P. Kuo A. Parry G. C. Shaw D. E. Callahan J. (2006). Structure of Human Urokinase Plasminogen Activator in Complex with its Receptor. Science 311 (5761), 656–659. 10.1126/science.1121143 16456079

[B79] Huttunen R. Syrjänen J. Vuento R. Hurme M. Huhtala H. Laine J. (2011). Plasma Level of Soluble Urokinase-type Plasminogen Activator Receptor as a Predictor of Disease Severity and Case Fatality in Patients with Bacteraemia: a Prospective Cohort Study. J. Intern. Med. 270 (1), 32–40. 10.1111/j.1365-2796.2011.02363.x 21332843

[B80] Inoshita H. Ohsawa I. Onda K. Tamano M. Horikoshi S. Ohi H. (2012). An Analysis of Functional Activity via the Three Complement Pathways during Hemodialysis Sessions: a New Insight into the Association between the Lectin Pathway and C5 Activation. Clin. Kidney J. 5 (5), 401–404. 10.1093/ckj/sfs089 26019815 PMC4432408

[B81] Ito T. Ichihashi T. Fujita H. Sugiura T. Ohte N. (2018). Impact of Malondialdehyde-Modified Low-Density Lipoprotein on Coronary Plaque Vulnerability in Patients Not Receiving Lipid-Lowering Therapy: a Whole Coronary Analysis with Multislice-Computed Tomography. Heart Vessels 33 (4), 351–357. 10.1007/s00380-017-1074-4 29067491

[B82] Jagiela J. Bartnicki P. Rysz J. (2020). Selected Cardiovascular Risk Factors in Early Stages of Chronic Kidney Disease. Int. Urol. Nephrol. 52 (2), 303–314. 10.1007/s11255-019-02349-1 31955363

[B83] Januzzi J. L. Pascual-Figal D. Daniels L. B. (2015). ST2 Testing for Chronic Heart Failure Therapy Monitoring: the International ST2 Consensus Panel. Am. J. Cardiol. 115 (7 Suppl. l), 70B–5B. 10.1016/j.amjcard.2015.01.044 25670638

[B84] Jhee J. H. Nam B. Y. Lee C. J. Park J. T. Han S. H. Kang S. W. (2021). Soluble Urokinase-type Plasminogen Activator Receptor, Changes of 24-Hour Blood Pressure, and Progression of Chronic Kidney Disease. J. Am. Heart Assoc. 10 (1), e017225. 10.1161/JAHA.120.017225 33325248 PMC7955457

[B85] Johansen K. L. Chertow G. M. Kutner N. G. Dalrymple L. S. Grimes B. A. Kaysen G. A. (2010). Low Level of Self-Reported Physical Activity in Ambulatory Patients New to Dialysis. Kidney Int. 78 (11), 1164–1170. 10.1038/ki.2010.312 20811334 PMC4170106

[B86] Johansen K. L. Garimella P. S. Hicks C. W. Kalra P. A. Kelly D. M. Martens S. (2021). Central and Peripheral Arterial Diseases in Chronic Kidney Disease: Conclusions from a Kidney Disease: Improving Global Outcomes (KDIGO) Controversies Conference. Kidney Int. 100 (1), 35–48. 10.1016/j.kint.2021.04.029 33961868 PMC9833277

[B87] Jun M. Venkataraman V. Razavian M. Cooper B. Zoungas S. Ninomiya T. (2012). Antioxidants for Chronic Kidney Disease. Cochrane Database Syst. Rev. 10, CD008176. 10.1002/14651858.CD008176.pub2 23076940 PMC8941641

[B88] Kalantar-Zadeh K. Kovesdy C. P. Streja E. Rhee C. M. Soohoo M. Chen J. L. T. (2017). Transition of Care from Pre-dialysis Prelude to Renal Replacement Therapy: the Blueprints of Emerging Research in Advanced Chronic Kidney Disease. Nephrol. Dial. Transpl. 32 (Suppl. l_2), ii91–ii98. 10.1093/ndt/gfw357 PMC5837675 28201698

[B89] Kalantar-Zadeh K. Moore L. W. Tortorici A. R. Chou J. A. St-Jules D. E. Aoun A. (2016). North American Experience with Low Protein Diet for Non-dialysis-dependent Chronic Kidney Disease. BMC Nephrol. 17 (1), 90. 10.1186/s12882-016-0304-9 27435088 PMC4952055

[B90] Kato A. Takita T. Furuhashi M. Maruyama Y. Hishida A. (2010). Comparison of Serum Albumin, C-Reactive Protein and Carotid Atherosclerosis as Predictors of 10-year Mortality in Hemodialysis Patients. Hemodial Int. 14 (2), 226–232. 10.1111/j.1542-4758.2009.00432.x 20345387

[B91] Kato S. Chmielewski M. Honda H. Pecoits-Filho R. Matsuo S. Yuzawa Y. (2008). Aspects of Immune Dysfunction in End-Stage Renal Disease. Clin. J. Am. Soc. Nephrol. 3 (5), 1526–1533. 10.2215/CJN.00950208 18701615 PMC4571158

[B92] Kim J. K. Lee H. W. Joo N. Lee H. S. Song Y. R. Kim H. J. (2020). Prognostic Role of Circulating Neutrophil Extracellular Traps Levels for Long-Term Mortality in New End-Stage Renal Disease Patients. Clin. Immunol. 210, 108263. 10.1016/j.clim.2019.108263 31629808

[B93] Kim Y. Molnar M. Z. Rattanasompattikul M. Hatamizadeh P. Benner D. Kopple J. D. (2013). Relative Contributions of Inflammation and Inadequate Protein Intake to Hypoalbuminemia in Patients on Maintenance Hemodialysis. Int. Urol. Nephrol. 45 (1), 215–227. 10.1007/s11255-012-0170-8 22528583 PMC4336193

[B94] Kishida K. Kishida N. Arima M. Nakatsuji H. Kobayashi H. Funahashi T. (2013). Serum C1q- Binding Adiponectin in Maintenance Hemodialysis Patients. BMC Nephrol. 14, 50. 10.1186/1471-2369-14-50 23442371 PMC3598349

[B95] Kisic B. Miric D. Dragojevic I. Rasic J. Popovic L. (2016). Role of Myeloperoxidase in Patients with Chronic Kidney Disease. Oxid Med. Cel Longev 2016, 1069743. 10.1155/2016/1069743 PMC4834151 27127544

[B96] Kloner R. A. (2020). Stunned and Hibernating Myocardium: Where Are We Nearly 4 Decades Later? J. Am. Heart Assoc. 9 (3), e015502. 10.1161/JAHA.119.015502 32013699 PMC7033879

[B97] Ko G. J. Kalantar-Zadeh K. Goldstein-Fuchs J. Rhee C. M. (2017). Dietary Approaches in the Management of Diabetic Patients with Kidney Disease. Nutrients 9 (8), 824. 10.3390/nu9080824 PMC5579617 28758978

[B98] Kooman J. P. Dekker M. J. Usvyat L. A. Kotanko P. van der Sande F. M. Schalkwijk C. G. (2017). Inflammation and Premature Aging in Advanced Chronic Kidney Disease. Am. J. Physiol. Ren. Physiol 313 (4), F938–F950. 10.1152/ajprenal.00256.2017 28701312

[B99] Kotanko P. Carter M. Levin N. W. (2006). Intestinal Bacterial Microflora-Aa Potential Source of Chronic Inflammation in Patients with Chronic Kidney Disease. Nephrol. Dial. Transpl. 21 (8), 2057–2060. 10.1093/ndt/gfl281 16762961

[B100] Kovesdy C. P. Kalantar-Zadeh K. (2009). Why Is Protein-Energy Wasting Associated with Mortality in Chronic Kidney Disease? Semin. Nephrol. 29 (1), 3–14. 10.1016/j.semnephrol.2008.10.002 19121469 PMC5500837

[B101] Krack A. Sharma R. Figulla H. R. Anker S. D. (2005). The Importance of the Gastrointestinal System in the Pathogenesis of Heart Failure. Eur. Heart J. 26 (22), 2368–2374. 10.1093/eurheartj/ehi389 15980032

[B102] Kraus D. von Jeinsen B. Tzikas S. Palapies L. Zeller T. Bickel C. (2018). Cardiac Troponins for the Diagnosis of Acute Myocardial Infarction in Chronic Kidney Disease. J. Am. Heart Assoc. 7 (19), e008032. 10.1161/JAHA.117.008032 30371308 PMC6404905

[B103] Kronenberg F. Lingenhel A. Neyer U. Lhotta K. König P. Auinger M. (2003). Prevalence of Dyslipidemic Risk Factors in Hemodialysis and CAPD Patients. Kidney Int. Suppl. 1 (84), S113–S116. 10.1046/j.1523-1755.63.s84.23.x 12694323

[B104] Lee S. R. Thorn S. Guerrera N. Gonzalez L. Taniguchi R. Langford J. (2021). Arteriovenous Fistula-Induced Cardiac Remodeling Shows Cardioprotective Features in Mice. JVS Vasc. Sci. 2, 110–128. 10.1016/j.jvssci.2021.05.002 34423320 PMC8375600

[B105] Liakopoulos V. Roumeliotis S. Gorny X. Dounousi E. Mertens P. R. (2017). Oxidative Stress in Hemodialysis Patients: A Review of the Literature. Oxid Med. Cel Longev 2017, 3081856. 10.1155/2017/3081856 PMC5613374 29138677

[B106] Liakopoulos V. Roumeliotis S. Zarogiannis S. Eleftheriadis T. Mertens P. R. (2019). Oxidative Stress in Hemodialysis: Causative Mechanisms, Clinical Implications, and Possible Therapeutic Interventions. Semin. Dial. 32 (1), 58–71. 10.1111/sdi.12745 30288786

[B107] Liberale L. Montecucco F. Schwarz L. Lüscher T. F. Camici G. G. (2021). Inflammation and Cardiovascular Diseases: Lessons from Seminal Clinical Trials. Cardiovasc. Res. 117 (2), 411–422. 10.1093/cvr/cvaa211 32666079

[B108] Liesa M. Shirihai O. S. (2013). Mitochondrial Dynamics in the Regulation of Nutrient Utilization and Energy Expenditure. Cell Metab 17 (4), 491–506. 10.1016/j.cmet.2013.03.002 23562075 PMC5967396

[B109] Lindner A. Charra B. Sherrard D. J. Scribner B. H. (1974). Accelerated Atherosclerosis in Prolonged Maintenance Hemodialysis. N. Engl. J. Med. 290 (13), 697–701. 10.1056/NEJM197403282901301 4813742

[B110] Locham S. Naazie I. Canner J. Siracuse J. Al-Nouri O. Malas M. (2021). Incidence and Risk Factors of Sepsis in Hemodialysis Patients in the United States. J. Vasc. Surg. 73 (3), 1016–1021. 10.1016/j.jvs.2020.06.126 32707386

[B111] Losappio V. Franzin R. Infante B. Godeas G. Gesualdo L. Fersini A. (2020). Molecular Mechanisms of Premature Aging in Hemodialysis: The Complex Interplay between Innate and Adaptive Immune Dysfunction. Int. J. Mol. Sci. 21 (10), 3422. 10.3390/ijms21103422 PMC7279398 32408613

[B112] Luczak M. Formanowicz D. Pawliczak E. Wanic-Kossowska M. Wykretowicz A. Figlerowicz M. (2011). Chronic Kidney Disease-Related Atherosclerosis - Proteomic Studies of Blood Plasma. Proteome Sci. 9, 25. 10.1186/1477-5956-9-25 21569504 PMC3112376

[B113] Lv L. Wang F. Wu L. Wang J. W. Cui Z. Hayek S. S. (2020). Soluble Urokinase-type Plasminogen Activator Receptor and Incident End-Stage Renal Disease in Chinese Patients with Chronic Kidney Disease. Nephrol. Dial. Transpl. 35 (3), 465–470. 10.1093/ndt/gfy265 PMC9214641 30124995

[B114] Ma K. W. Greene E. L. Raij L. (1992). Cardiovascular Risk Factors in Chronic Renal Failure and Hemodialysis Populations. Am. J. Kidney Dis. 19 (6), 505–513. 10.1016/s0272-6386(12)80827-4 1534442

[B115] Madero M. Sarnak M. J. Wang X. Greene T. Beck G. J. Kusek J. W. (2009). Uric Acid and Long-Term Outcomes in CKD. Am. J. Kidney Dis. 53 (5), 796–803. 10.1053/j.ajkd.2008.12.021 19303683 PMC2691553

[B116] Major R. W. Oozeerally I. Dawson S. Riddleston H. Gray L. J. Brunskill N. J. (2016). Aspirin and Cardiovascular Primary Prevention in Non-endstage Chronic Kidney Disease: A Meta-Analysis. Atherosclerosis 251, 177–182. 10.1016/j.atherosclerosis.2016.06.013 27341534

[B117] Marants R. Qirjazi E. Lai K. B. Szeto C. C. Li P. K. T. Li F. (2021). Exploring the Link between Hepatic Perfusion and Endotoxemia in Hemodialysis. Kidney Int. Rep. 6 (5), 1336–1345. 10.1016/j.ekir.2021.02.008 34013112 PMC8116762

[B118] Mares J. Richtrova P. Hricinova A. Tuma Z. Moravec J. Lysak D. (2010). Proteomic Profiling of Blood-Dialyzer Interactome Reveals Involvement of Lectin Complement Pathway in Hemodialysis-Induced Inflammatory Response. Proteomics Clin. Appl. 4 (10-11), 829–838. 10.1002/prca.201000031 21137026

[B119] Mares J. Thongboonkerd V. Tuma Z. Moravec J. Matejovic M. (2009). Specific Adsorption of Some Complement Activation Proteins to Polysulfone Dialysis Membranes during Hemodialysis. Kidney Int. 76 (4), 404–413. 10.1038/ki.2009.138 19421191

[B120] Martin C. Burdon P. C. Bridger G. Gutierrez-Ramos J. C. Williams T. J. Rankin S. M. (2003). Chemokines Acting via CXCR2 and CXCR4 Control the Release of Neutrophils from the Bone Marrow and Their Return Following Senescence. Immunity 19 (4), 583–593. 10.1016/s1074-7613(03)00263-2 14563322

[B121] Martínez-Klimova E. Aparicio-Trejo O. E. Gómez-Sierra T. Jiménez-Uribe A. P. Bellido B. Pedraza-Chaverri J. (2020). Mitochondrial Dysfunction and Endoplasmic Reticulum Stress in the Promotion of Fibrosis in Obstructive Nephropathy Induced by Unilateral Ureteral Obstruction. Biofactors 46 (5), 716–733. 10.1002/biof.1673 32905648

[B122] Mayer K. Bernlochner I. Braun S. Schulz S. Orban M. Morath T. (2014). Aspirin Treatment and Outcomes after Percutaneous Coronary Intervention: Results of the ISAR-ASPI Registry. J. Am. Coll. Cardiol. 64 (9), 863–871. 10.1016/j.jacc.2014.05.049 25169169

[B123] McIntyre C. W. Harrison L. E. Eldehni M. T. Jefferies H. J. Szeto C. C. John S. G. (2011). Circulating Endotoxemia: a Novel Factor in Systemic Inflammation and Cardiovascular Disease in Chronic Kidney Disease. Clin. J. Am. Soc. Nephrol. 6 (1), 133–141. 10.2215/CJN.04610510 20876680 PMC3022234

[B124] Miller-Hodges E. Anand A. Shah A. S. V. Chapman A. R. Gallacher P. Lee K. K. (2018). High-Sensitivity Cardiac Troponin and the Risk Stratification of Patients with Renal Impairment Presenting with Suspected Acute Coronary Syndrome. Circulation 137 (5), 425–435. 10.1161/CIRCULATIONAHA.117.030320 28978551 PMC5793996

[B125] Mirna M. Topf A. Wernly B. Rezar R. Paar V. Jung C. (2020). Novel Biomarkers in Patients with Chronic Kidney Disease: An Analysis of Patients Enrolled in the GCKD-Study. J. Clin. Med. 9 (3), 886. 10.3390/jcm9030886 PMC7141541 32213894

[B126] Moore M. A. Kaplan D. S. Picciolo G. L. Wallis R. R. Kowolik M. J. (2001). Effect of Cellulose Acetate Materials on the Oxidative Burst of Human Neutrophils. J. Biomed. Mater. Res. 55 (3), 257–265. 10.1002/1097-4636(20010605)55:3<257:aid-jbm1013>3.0.co;2-h 11255178

[B127] Mühl H. Sandau K. Brüne B. Briner V. A. Pfeilschifter J. (1996). Nitric Oxide Donors Induce Apoptosis in Glomerular Mesangial Cells, Epithelial Cells and Endothelial Cells. Eur. J. Pharmacol. 317 (1), 137–149. 10.1016/s0014-2999(96)00701-7 8982730

[B128] Mun K. C. Golper T. A. (2000). Impaired Biological Activity of Erythropoietin by Cyanate Carbamylation. Blood Purif. 18 (1), 13–17. 10.1159/000014403 10686438

[B129] Nakayama M. Tani Y. Zhu W. J. Watanabe K. Yokoyama K. Fukagawa M. (2018). Oral Ferric Citrate Hydrate Associated with Less Oxidative Stress Than Intravenous Saccharated Ferric Oxide. Kidney Int. Rep. 3 (2), 364–373. 10.1016/j.ekir.2017.10.016 29725640 PMC5932126

[B130] Nishizawa Y. Shoji T. Kakiya R. Tsujimoto Y. Tabata T. Ishimura E. (2003). Non-high-density Lipoprotein Cholesterol (Non-HDL-C) as a Predictor of Cardiovascular Mortality in Patients with End-Stage Renal Disease. Kidney Int. Suppl. 1 (84), S117–S120. 10.1046/j.1523-1755.63.s84.30.x 12694324

[B131] Oberg B. P. McMenamin E. Lucas F. L. McMonagle E. Morrow J. Ikizler T. A. (2004). Increased Prevalence of Oxidant Stress and Inflammation in Patients with Moderate to Severe Chronic Kidney Disease. Kidney Int. 65 (3), 1009–1016. 10.1111/j.1523-1755.2004.00465.x 14871421

[B132] Papagianni A. Kalovoulos M. Kirmizis D. Vainas A. Belechri A. M. Alexopoulos E. (2003). Carotid Atherosclerosis Is Associated with Inflammation and Endothelial Cell Adhesion Molecules in Chronic Haemodialysis Patients. Nephrol. Dial. Transpl. 18 (1), 113–119. 10.1093/ndt/18.1.113 12480968

[B133] Pappas E. M. Mpournaka S. Katopodis P. Chardalias A. Tsakas S. Theodoros T. (2019). The Effect of Dialysis Modality and Membrane Performance on Native Immunity in Dialysis Patients. Pril (Makedon Akad Nauk Umet Odd Med Nauki) 40 (2), 25–32. 10.2478/prilozi-2019-0011 31605588

[B134] Pawlak K. Mysliwiec M. Pawlak D. (2013). Oxidized Low-Density Lipoprotein (oxLDL) Plasma Levels and oxLDL to LDL Ratio - Are They Real Oxidative Stress Markers in Dialyzed Patients? Life Sci. 92 (4-5), 253–258. 10.1016/j.lfs.2012.12.002 23295961

[B135] Pawlak K. Pawlak D. Mysliwiec M. (2007). Impaired Renal Function and Duration of Dialysis Therapy Are Associated with Oxidative Stress and Proatherogenic Cytokine Levels in Patients with End-Stage Renal Disease. Clin. Biochem. 40 (1-2), 81–85. 10.1016/j.clinbiochem.2006.09.001 17046733

[B136] Pecoits-Filho R. Bárány P. Lindholm B. Heimbürger O. Stenvinkel P. (2002). Interleukin-6 Is an Independent Predictor of Mortality in Patients Starting Dialysis Treatment. Nephrol. Dial. Transpl. 17 (9), 1684–1688. 10.1093/ndt/17.9.1684 12198224

[B137] Pencak P. Czerwieńska B. Ficek R. Wyskida K. Kujawa-Szewieczek A. Olszanecka-Glinianowicz M. (2013). Calcification of Coronary Arteries and Abdominal Aorta in Relation to Traditional and Novel Risk Factors of Atherosclerosis in Hemodialysis Patients. BMC Nephrol. 14, 10. 10.1186/1471-2369-14-10 23317172 PMC3556324

[B138] Pertosa G. Tarantino E. A. Gesualdo L. Montinaro V. Schena F. P. (1993). C5b-9 Generation and Cytokine Production in Hemodialyzed Patients. Kidney Int. Suppl. 41, S221–S225. 8320926

[B139] Podkowinska A. Formanowicz D. (2020). Chronic Kidney Disease as Oxidative Stress- and Inflammatory-Mediated Cardiovascular Disease. Antioxidants (Basel) 9 (8). 10.3390/antiox9080752 PMC7463588 32823917

[B140] Polzin A. Dannenberg L. Sansone R. Levkau B. Kelm M. Hohlfeld T. (2016). Antiplatelet Effects of Aspirin in Chronic Kidney Disease Patients. J. Thromb. Haemost. 14 (2), 375–380. 10.1111/jth.13211 26644261

[B141] Ponikowski P. Voors A. A. Anker S. D. Bueno H. Cleland J. G. Coats A. J. (20162016). 2016 ESC Guidelines for the Diagnosis and Treatment of Acute and Chronic Heart Failure: The Task Force for the Diagnosis and Treatment of Acute and Chronic Heart Failure of the European Society of Cardiology (ESC). Developed with the Special Contribution of the Heart Failure Association (HFA) of the ESC. Eur. J. Heart Fail. 18 (27), 891–975. 10.1002/ejhf.592 27207191

[B142] Poppelaars F. Faria B. Gaya da Costa M. Franssen C. F. M. van Son W. J. Berger S. P. (2018). The Complement System in Dialysis: A Forgotten Story? Front. Immunol. 9, 71. 10.3389/fimmu.2018.00071 29422906 PMC5788899

[B143] Poulianiti K. P. Kaltsatou A. Mitrou G. I. Jamurtas A. Z. Koutedakis Y. Maridaki M. (2016). Systemic Redox Imbalance in Chronic Kidney Disease: A Systematic Review. Oxid Med. Cel Longev 2016, 8598253. 10.1155/2016/8598253 PMC4987477 27563376

[B144] Pradhan A. D. Aday A. W. Rose L. M. Ridker P. M. (2018). Residual Inflammatory Risk on Treatment with PCSK9 Inhibition and Statin Therapy. Circulation 138 (2), 141–149. 10.1161/CIRCULATIONAHA.118.034645 29716940 PMC8108606

[B145] Raber I. McCarthy C. P. Januzzi J. L. Jr. (2021). A Test in Context: Interpretation of High-Sensitivity Cardiac Troponin Assays in Different Clinical Settings. J. Am. Coll. Cardiol. 77 (10), 1357–1367. 10.1016/j.jacc.2021.01.011 33706879

[B146] Raber I. McCarthy C. P. Januzzi J. L. (2021). A Test in Context: Interpretation of High-Sensitivity Cardiac Troponin Assays in Different Clinical Settings. J. Am. Coll. Cardiol. 77 (10), 1357–1367. 10.1016/j.jacc.2021.01.011 33706879

[B147] Rapa S. F. Prisco F. Popolo A. Iovane V. Autore G. Di Iorio B. R. (2021). Pro-Inflammatory Effects of Indoxyl Sulfate in Mice: Impairment of Intestinal Homeostasis and Immune Response. Int. J. Mol. Sci. 22 (3). 10.3390/ijms22031135 PMC7865799 33498967

[B148] Rayego-Mateos S. Valdivielso J. M. (2020). New Therapeutic Targets in Chronic Kidney Disease Progression and Renal Fibrosis. Expert Opin. Ther. Targets 24 (7), 655–670. 10.1080/14728222.2020.1762173 32338087

[B149] Ridker P. M. Everett B. M. Thuren T. MacFadyen J. G. Chang W. H. Ballantyne C. (2017). Antiinflammatory Therapy with Canakinumab for Atherosclerotic Disease. N. Engl. J. Med. 377 (12), 1119–1131. 10.1056/NEJMoa1707914 28845751

[B150] Ridker P. M. MacFadyen J. G. Glynn R. J. Koenig W. Libby P. Everett B. M. (2018). Inhibition of Interleukin-1β by Canakinumab and Cardiovascular Outcomes in Patients with Chronic Kidney Disease. J. Am. Coll. Cardiol. 71 (21), 2405–2414. 10.1016/j.jacc.2018.03.490 29793629

[B151] Rocha S. Valente M. J. Coimbra S. Catarino C. Rocha-Pereira P. Oliveira J. G. (2021). Interleukin 6 (Rs1800795) and Pentraxin 3 (Rs2305619) Polymorphisms-Association with Inflammation and All-Cause Mortality in End-Stage-Renal Disease Patients on Dialysis. Sci. Rep. 11 (1), 14768. 10.1038/s41598-021-94075-x 34285273 PMC8292348

[B152] Rotbain Curovic V. Theilade S. Winther S. A. Tofte N. Eugen-Olsen J. Persson F. (2019). Soluble Urokinase Plasminogen Activator Receptor Predicts Cardiovascular Events, Kidney Function Decline, and Mortality in Patients with Type 1 Diabetes. Diabetes care 42 (6), 1112–1119. 10.2337/dc18-1427 30885954

[B153] Rothschild M. A. Oratz M. Schreiber S. S. (1973). Albumin Metabolism. Gastroenterology 64 (2), 324–337. 10.1016/s0016-5085(73)80046-0 4568596

[B154] Rousseau Y. Carreno M. P. Poignet J. L. Kazatchkine M. D. Haeffner-Cavaillon N. (1999). Dissociation between Complement Activation, Integrin Expression and Neutropenia during Hemodialysis. Biomaterials 20 (20), 1959–1967. 10.1016/s0142-9612(99)00101-5 10514074

[B155] Rysz J. Franczyk B. Ławiński J. Olszewski R. Ciałkowska-Rysz A. Gluba-Brzózka A. (2021). The Impact of CKD on Uremic Toxins and Gut Microbiota. Toxins (Basel) 13 (4), 252. 10.3390/toxins13040252 33807343 PMC8067083

[B156] Rysz J. Franczyk B. Ławiński J. Olszewski R. Ciałkowska-Rysz A. Gluba-Brzózka A. J. T. (2021). The Impact of CKD on Uremic Toxins and Gut Microbiota. Toxins (Basel) 13 (4), 252. 10.3390/toxins13040252 33807343 PMC8067083

[B157] Sasaki K. Shoji T. Kabata D. Shintani A. Okute Y. Tsuchikura S. (2021). Oxidative Stress and Inflammation as Predictors of Mortality and Cardiovascular Events in Hemodialysis Patients: The DREAM Cohort. J. Atheroscler. Thromb. 28 (3), 249–260. 10.5551/jat.56069 32741893 PMC8049144

[B158] Schettler V. Wieland E. Methe H. Schuff-Werner P. Müller G. A. (1998). Oxidative Stress during Dialysis: Effect on Free Radical Scavenging Enzyme (FRSE) Activities and Glutathione (GSH) Concentration in Granulocytes. Nephrol. Dial. Transpl. 13 (10), 2588–2593. 10.1093/ndt/13.10.2588 9794565

[B159] Sedeek M. Nasrallah R. Touyz R. M. Hébert R. L. (2013). NADPH Oxidases, Reactive Oxygen Species, and the Kidney: Friend and Foe. J. Am. Soc. Nephrol. 24 (10), 1512–1518. 10.1681/ASN.2012111112 23970124 PMC3785272

[B160] Sepe V. Gregorini M. Rampino T. Esposito P. Coppo R. Galli F. (2019). Vitamin E-Loaded Membrane Dialyzers Reduce Hemodialysis Inflammaging. BMC Nephrol. 20 (1), 412. 10.1186/s12882-019-1585-6 31729973 PMC6858730

[B161] Shahzad K. Bock F. Dong W. Wang H. Kopf S. Kohli S. (2015). Nlrp3-inflammasome Activation in Non-myeloid-derived Cells Aggravates Diabetic Nephropathy. Kidney Int. 87 (1), 74–84. 10.1038/ki.2014.271 25075770 PMC4284813

[B162] Shoji T. Nishizawa Y. Kawagishi T. Kawasaki K. Taniwaki H. Tabata T. (1998). Intermediate-density Lipoprotein as an Independent Risk Factor for Aortic Atherosclerosis in Hemodialysis Patients. J. Am. Soc. Nephrol. 9 (7), 1277–1284. 10.1681/ASN.V971277 9644639

[B163] Shuai T. Yan P. Xiong H. Huang Q. Zhu L. Yang K. (2019). Association between Soluble Urokinase-type Plasminogen Activator Receptor Levels and Chronic Kidney Disease: A Systematic Review and Meta-Analysis. Biomed. Res. Int. 2019, 6927456. 10.1155/2019/6927456 31886242 PMC6899318

[B164] Siems W. Quast S. Carluccio F. Wiswedel I. Hirsch D. Augustin W. (2002). Oxidative Stress in Chronic Renal Failure as a Cardiovascular Risk Factor. Clin. Nephrol. 58 (Suppl. 1), S12–S19. 12227720

[B165] Simone S. Rascio F. Castellano G. Divella C. Chieti A. Ditonno P. (2014). Complement-dependent NADPH Oxidase Enzyme Activation in Renal Ischemia/reperfusion Injury. Free Radic. Biol. Med. 74, 263–273. 10.1016/j.freeradbiomed.2014.07.003 25017967

[B166] Spoto B. Mattace-Raso F. Sijbrands E. Leonardis D. Testa A. Pisano A. (2015). Association of IL-6 and a Functional Polymorphism in the IL-6 Gene with Cardiovascular Events in Patients with CKD. Clin. J. Am. Soc. Nephrol. 10 (2), 232–240. 10.2215/CJN.07000714 25492254 PMC4317745

[B167] Stenvinkel P. Ketteler M. Johnson R. J. Lindholm B. Pecoits-Filho R. Riella M. (2005). IL-10, IL-6, and TNF-Alpha: central Factors in the Altered Cytokine Network of Uremia-Tthe Good, the Bad, and the Ugly. Kidney Int. 67 (4), 1216–1233. 10.1111/j.1523-1755.2005.00200.x 15780075

[B168] Strålberg T. Nordenskjöld A. Cao Y. Kublickiene K. Nilsson E. (2019). Proprotein Convertase Subtilisin/kexin Type 9 and Mortality in Patients Starting Hemodialysis. Eur. J. Clin. Invest. 49 (7), e13113. 30921469 10.1111/eci.13113

[B169] Sun J. Axelsson J. Machowska A. Heimbürger O. Bárány P. Lindholm B. (2016). Biomarkers of Cardiovascular Disease and Mortality Risk in Patients with Advanced CKD. Clin. J. Am. Soc. Nephrol. 11 (7), 1163–1172. 10.2215/CJN.10441015 27281698 PMC4934843

[B170] Tetta C. Biasioli S. Schiavon R. Inguaggiato P. David S. Panichi V. (1999). An Overview of Haemodialysis and Oxidant Stress. Blood Purif. 17 (2-3), 118–126. 10.1159/000014383 10449869

[B171] Thang L. V. Loc N. D. Kien N. T. Dung N. H. Quyen D. B. Q. Tuan N. M. (2020). Interleukin 6 Is a Better Predictor of 5-year Cardiovascular Mortality Than High-Sensitivity C-Reactive Protein in Hemodialysis Patients Using Reused Low-Flux Dialyzers. Int. Urol. Nephrol. 52 (6), 1135–1142. 10.1007/s11255-020-02461-7 32306196

[B172] Tsai M. Liou H. Huang Y. Lee T. Chen M. Fang Y. (2021). Hazardous Effect of Low-Dose Aspirin in Patients with Predialysis Advanced Chronic Kidney Disease Assessed by Machine Learning Method Feature Selection. Healthcare (Basel, Switzerland) 9 (11), 1484. 10.3390/healthcare9111484 PMC8625790 34828530

[B173] Tsuchikura S. Shoji T. Shimomura N. Kakiya R. Emoto M. Koyama H. (2010). Serum C-Reactive Protein and Thioredoxin Levels in Subjects with Mildly Reduced Glomerular Filtration Rate. BMC Nephrol. 11, 7. 10.1186/1471-2369-11-7 20423474 PMC2868841

[B174] Twerenbold R. Badertscher P. Boeddinghaus J. Nestelberger T. Wildi K. Puelacher C. (2018). 0/1-Hour Triage Algorithm for Myocardial Infarction in Patients with Renal Dysfunction. Circulation 137 (5), 436–451. 10.1161/CIRCULATIONAHA.117.028901 29101287 PMC5794234

[B175] Unger E. D. Dubin R. F. Deo R. Daruwalla V. Friedman J. L. Medina C. (2016). Association of Chronic Kidney Disease with Abnormal Cardiac Mechanics and Adverse Outcomes in Patients with Heart Failure and Preserved Ejection Fraction. Eur. J. Heart Fail. 18 (1), 103–112. 10.1002/ejhf.445 26635076 PMC4713321

[B176] Valdivielso J. M. Rodríguez-Puyol D. Pascual J. Barrios C. Bermúdez-López M. Sánchez-Niño M. D. (2019). Atherosclerosis in Chronic Kidney Disease: More, Less, or Just Different? Arterioscler Thromb. Vasc. Biol. 39 (10), 1938–1966. 10.1161/ATVBAHA.119.312705 31412740

[B177] Valga F. Monzon T. Vega-Diaz N. Rodriguez-Perez J. C. Ruiz-Santana S. (2021). Inflammation and Hemodialysis Adequacy: Are C-Reactive Protein Levels Influenced by Dialysis Dose? Nefrologia S0211-6995 (21), 00112–120. 10.1016/j.nefro.2021.06.001 36153912

[B178] Valtuille R. A. Rossi G. Gimenez E. (2021). Protective Effect of Autologous Arteriovenous Fistulae against Oxidative Stress in Hemodialyzed Patients. Cureus 13 (6), e15398. 10.7759/cureus.15398 34249547 PMC8253232

[B179] van Rosendael A. R. van den Hoogen I. J. Gianni U. Ma X. Tantawy S. W. Bax A. M. (2021). Association of Statin Treatment with Progression of Coronary Atherosclerotic Plaque Composition. JAMA Cardiol. 6 (11), 1257–1266. 10.1001/jamacardio.2021.3055 34406326 PMC8374741

[B180] Varan H. I. Dursun B. Dursun E. Ozben T. Suleymanlar G. (2010). Acute Effects of Hemodialysis on Oxidative Stress Parameters in Chronic Uremic Patients: Comparison of Two Dialysis Membranes. Int. J. Nephrol. Renovasc Dis. 3, 39–45. 10.2147/ijnrd.s6598 21694927 PMC3108776

[B181] Vaziri N. D. Dicus M. Ho N. D. Boroujerdi-Rad L. Sindhu R. K. (2003). Oxidative Stress and Dysregulation of Superoxide Dismutase and NADPH Oxidase in Renal Insufficiency. Kidney Int. 63 (1), 179–185. 10.1046/j.1523-1755.2003.00702.x 12472781

[B182] Vlad C. Foia L. Pavel-Tanasa M. Toma V. Florea L. Voroneanu L. (2021). Evaluation of Cardiovascular Events and Progression to End-Stage Renal Disease in Patients with Dyslipidemia and Chronic Kidney Disease from the North-Eastern Area of Romania. Int. Urol. Nephrol. 1, 1. 10.1007/s11255-021-02919-2 34224064

[B183] Wan C. Su H. Zhang C. (2016). Role of NADPH Oxidase in Metabolic Disease-Related Renal Injury: An Update. Oxid Med. Cel Longev 2016, 7813072. 10.1155/2016/7813072 PMC5002489 27597884

[B184] Wei C. El Hindi S. Li J. Fornoni A. Goes N. Sageshima J. (2011). Circulating Urokinase Receptor as a Cause of Focal Segmental Glomerulosclerosis. Nat. Med. 17 (8), 952–960. 10.1038/nm.2411 21804539 PMC4089394

[B185] Westerweel P. E. Hoefer I. E. Blankestijn P. J. de Bree P. Groeneveld D. van Oostrom O. (2007). End-stage Renal Disease Causes an Imbalance between Endothelial and Smooth Muscle Progenitor Cells. Am. J. Physiol. Ren. PhysiolRenal Physiol. 292 (4), F1132–F1140. 10.1152/ajprenal.00163.2006 17200161

[B186] Wherry E. J. Kurachi M. (2015). Molecular and Cellular Insights into T Cell Exhaustion. Nat. Rev. Immunol. 15 (8), 486–499. 10.1038/nri3862 26205583 PMC4889009

[B187] Witko-Sarsat V. Friedlander M. Nguyen Khoa T. Capeillère-Blandin C. Nguyen A. T. Canteloup S. (1998). Advanced Oxidation Protein Products as Novel Mediators of Inflammation and Monocyte Activation in Chronic Renal Failure. J. Immunol. 161 (5), 2524–2532. 9725252

[B188] Wolfe R. Wetmore J. B. Woods R. L. McNeil J. J. Gallagher H. Roderick P. (2021). Subgroup Analysis of the ASPirin in Reducing Events in the Elderly Randomized Clinical Trial Suggests Aspirin Did Not Improve Outcomes in Older Adults with Chronic Kidney Disease. Kidney Int. 99 (2), 466–474. 10.1016/j.kint.2020.08.011 32920022 PMC7957958

[B189] Yamazaki T. Froelicher V. F. Myers J. Chun S. Wang P. (2005). Spatial QRS-T Angle Predicts Cardiac Death in a Clinical Population. Heart Rhythm 2 (1), 73–78. 10.1016/j.hrthm.2004.10.040 15851268

[B190] Yang R. B. Mark M. R. Gray A. Huang A. Xie M. H. Zhang M. (1998). Toll-like Receptor-2 Mediates Lipopolysaccharide-Induced Cellular Signalling. Nature 395 (6699), 284–288. 10.1038/26239 9751057

[B191] Yoon J. W. Pahl M. V. Vaziri N. D. (2007). Spontaneous Leukocyte Activation and Oxygen-free Radical Generation in End-Stage Renal Disease. Kidney Int. 71 (2), 167–172. 10.1038/sj.ki.5002019 17136029

[B192] Yu A. W. Nawab Z. M. Barnes W. E. Lai K. N. Ing T. S. Daugirdas J. T. (1997). Splanchnic Erythrocyte Content Decreases during Hemodialysis: a New Compensatory Mechanism for Hypovolemia. Kidney Int. 51 (6), 1986–1990. 10.1038/ki.1997.270 9186892

[B193] Yu T. M. Lin C. L. Shu K. H. Liu Y. L. Chen C. H. Huang S. T. (2015). Increased Risk of Cardiovascular Events in End-Stage Renal Disease Patients with Osteoporosis: a Nationwide Population-Based Cohort Study. Osteoporos. Int. 26 (2), 785–793. 10.1007/s00198-014-2982-0 25491767

[B194] Zhang D. W. Lagace T. A. Garuti R. Zhao Z. McDonald M. Horton J. D. (2007). Binding of Proprotein Convertase Subtilisin/kexin Type 9 to Epidermal Growth Factor-like Repeat A of Low Density Lipoprotein Receptor Decreases Receptor Recycling and Increases Degradation. J. Biol. Chem. 282 (25), 18602–18612. 10.1074/jbc.M702027200 17452316

[B195] Zhang H. Fan L. Liao H. Tu L. Zhang J. Xu D. (2021). Correlations of Cardiac Function with Inflammation, Oxidative Stress and Anemia in Patients with Uremia. Exp. Ther. Med. 21 (3), 250. 10.3892/etm.2021.9681 33603858 PMC7851606

[B196] Zhao W. M. Tao S. M. Liu G. L. (2020). Neutrophil-to-lymphocyte Ratio in Relation to the Risk of All-Cause Mortality and Cardiovascular Events in Patients with Chronic Kidney Disease: a Systematic Review and Meta-Analysis. Ren. Fail. 42 (1), 1059–1066. 10.1080/0886022X.2020.1832521 33081569 PMC7668415

[B197] Zhou M. Du Y. Wu Y. Zhang P. Liu P. Li J. (2021). Analysis of Inflammatory Factor Levels in Serum and Risk Factors in Patients with Chronic Renal Failure Undergoing Maintenance Hemodialysis. Am. J. Transl Res. 13 (6), 6994–7000. 34306454 PMC8290784

[B198] Ziegler D. V. Wiley C. D. Velarde M. C. (2015). Mitochondrial Effectors of Cellular Senescence: beyond the Free Radical Theory of Aging. Aging Cell 14 (1), 1–7. 10.1111/acel.12287 25399755 PMC4310776

[B199] Zoccali C. Moissl U. Chazot C. Mallamaci F. Tripepi G. Arkossy O. (2017). Chronic Fluid Overload and Mortality in ESRD. J. Am. Soc. Nephrol. 28 (8), 2491–2497. 10.1681/ASN.2016121341 28473637 PMC5533242

[B200] Zuidema M. Y. Dellsperger K. C. (2012). Myocardial Stunning with Hemodialysis: Clinical Challenges of the Cardiorenal Patient. Cardiorenal Med. 2 (2), 125–133. 10.1159/000337476 22851961 PMC3376339

